# Triterpenoids from *Cyclocarya paliurus* that Enhance Glucose Uptake in 3T3-L1 Adipocytes

**DOI:** 10.3390/molecules24010187

**Published:** 2019-01-06

**Authors:** Zhu-Jun Fang, Sheng-Nan Shen, Jia-Min Wang, Yong-Jiang Wu, Chang-Xin Zhou, Jian-Xia Mo, Li-Gen Lin, Li-She Gan

**Affiliations:** 1 College of Pharmaceutical Sciences, Zhejiang University, 866 Yuhangtang Road, Hangzhou 310058, China; 11519027@zju.edu.cn (Z.-J.F.); 21819075@zju.edu.cn (J.-M.W.); yjwu@zju.edu.cn (Y.-J.W.); zhoucx10@zju.edu.cn (C.-X.Z.); mojx@zju.edu.cn (J.-X.M.); 2 State Key Laboratory of Quality Research in Chinese Medicine, Institute of Chinese Medical Sciences, University of Macau, Macao 999078, China; yb57518@um.edu.mo; 3 Hangzhou Institute of Innovative Medicine, Zhejiang University, 291 Fucheng Road, Hangzhou 310018, China

**Keywords:** *Cyclocarya paliurus* (Juglandaceae), *seco*-dammarane triterpenoids, glucose uptake, adipocytes

## Abstract

Four previously undescribed compounds, including three rarely occurring *seco*-dammarane triterpenoid glycosides and a pentacyclic triterpenic acid, were isolated from a 70% ethanol extract of the leaves of *Cyclocarya paliurus* (Juglandaceae), along with eleven known triterpenoids. Their structures were determined by spectroscopic techniques, including 2D NMR and HRESIMS, as well as chemical methods. Among them, several triterpenoids enhanced insulin stimulated glucose uptake in both 3T3-L1 adipocytes and C2C12 myotubes. Furthermore, compound **1** dose-dependently increased glucose uptake through activating AMP-activated protein kinase (AMPK)-p38 pathway. Collectively, triterpenoids from *C. paliurus* could be developed as insulin sensitizers, which might have therapeutic potential for insulin resistance and hyperglycemia.

## 1. Introduction

Long-term use of oral hypoglycemic agents or insulin is accompanied by side effects, including hypoglycemic episodes, edema, hepatorenal disturbances, and gastrointestinal problems [1,2]. Due to their perceived better safety and efficacy, phytochemicals such as phenolic acids, flavonoids, alkaloids, polysaccharides, and triterpenoids have attracted more and more attention in the treatment of diabetes [3]. Many triterpenoids have been revealed to possess potential therapeutical effects to treat diabetes. Cucurbitane-type triterpenoids were reported to improve insulin sensitivity and glucose homeostasis in C2C12 myotubes and a streptozotocin (STZ)-induced diabetic mouse model [4]. Nanoencapsulated triterpenoids from Petri dish-cultured *Antrodia cinnamomea* ameliorate hyperglycemia on high-fat diet (HFD)- and STZ-induced diabetic rats [5]. The extract of a triterpenoid-enriched Jamun fruit attenuates hyperglycemia and glucose intolerance, prevents the abnormal elevation of hepatic gluconeogenesis, and improves dyslipidemia in STZ-induced diabetic mice [6]. Ursolic acid was evaluated to improve insulin sensitivity and promote glucose uptake and utilization [7,8].

*Cyclocarya paliurus* (Batalin) Iljinsk., a Chinese endemic plant, belongs to the family Juglandaceae and is mainly distributed in South and Southeast China [9]. Its leaves have been used as folk medicines for the prevention and treatment of diabetes mellitus, hypertension and dyslipidemia [10] and approved as a new food material by the China FDA in 2014. To date, triterpenoids, flavonoids, phenolic acids, and polysaccharides have been isolated from this plant [11,12,13,14]. Previous studies revealed that *C. paliurus* ethanol extract exhibits antihyperglycemic effects, and ameliorates insulin resistance in type 2 diabetic rats [15,16], in which triterpenoids might be the major functional components [17,18]. For example, cyclocaric acid B and cyclocarioside H promote glucose uptake in the absence of insulin, as well as ameliorate insulin receptor substrate 1 (IRS-1)/phosphoinositide 3-kinase (PI3K)/protein kinase B (Akt) pathway by inhibiting inflammation in 3T3-L1 adipocytes [19].

**Figure 1:**
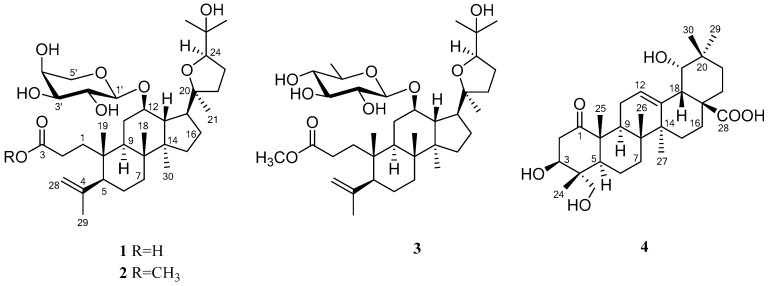
Chemical structures of compounds **1**–**4**.

In a continuing search for insulin sensitizing compounds from *C. paliurus*, four undescribed compounds **1**–**4** (Figure 1), including three rarely occurring *seco*-dammarane triterpenoid glycosides **1**–**3** possessing 20,24-epoxy linkages, and a pentacyclic triterpenic acid **4**, were systematically isolated, along with eleven known triterpenoids **5**–**15**. Their structures were elucidated on the basis of chemical and spectroscopic approaches, including 2D NMR and HRESIMS techniques. Finally the effects of the isolates in insulin-stimulated glucose uptake in 3T3-L1 adipocytes and C2C12 myotubes were further evaluated.

## 2. Results and Discussion

### 2.1. Identification of New Compounds

**Figure 2:**
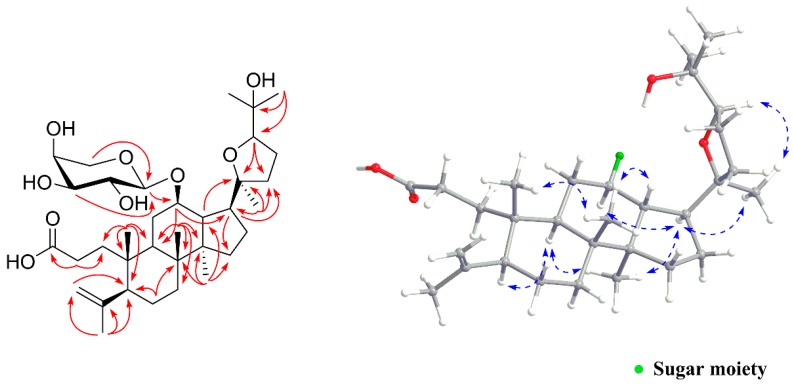
Key HMBC (red arrows) and NOESY (blue dotted double arrows) correlations of **1**.

Compound **1** was isolated as a white amorphous powder, which had a molecular formula of C_35_H_58_O_9_ as deduced from a HRESIMS pseudo ion peak at 645.3978 [M + Na]^+^ (calcd. for C_35_H_58_O_9_Na, 645.3979). The IR spectrum showed absorption bands for carboxyl (1699 cm^−1^ and 3405 cm^−1^) and double bond (1685 cm^−1^) functions. The ^1^H-NMR spectrum (Table 1) of **1** exhibited seven methyl singlets at δ_H_ 1.00 (s, Me-30), 1.77 (s, Me-29), 1.16 (s, Me-27), 1.19 (s, Me-26), 1.16 (s, Me-21), 1.10 (s, Me-19), and 1.06 (s, Me-18), as well as terminal methylene olefinic protons at δ_H_ 4.72 (d, *J* = 1.4 Hz, H-28a) and 4.85 (d, *J* = 1.4 Hz, H-28b). The ^13^C-NMR (Table 1) and DEPT (135) spectra revealed a carboxyl group at δ_C_ 180.2, one terminal double bond at δ_C_ 149.3 and 114.1, and four oxygen-bearing carbons at δ_C_ 76.5 (C-12), 87.8 (C-20), 84.9 (C-24), and 72.7 (C-25). Besides, the presence of an anomeric hydrogen at δ_H_ 4.29 (d, *J* = 7.1 Hz, H-1′) in the ^1^H-NMR spectrum and five carbon signals at δ_C_ 101.2, 72.8, 74.6, 70.4, and 67.8 indicated an arabinosyl moiety. The above data suggested an unusual 3,4-*seco*-dammarane type triterpenoid glycoside structurefor **1**, similar to that of the known compound cyclocarioside F [14], except for signals of the side-chain. In the HMBC spectrum (Figure 2), the *seco*-dammarane skeleton was confirmed by correlations from H_3_-29 to C-4, C-5, and C-28, from H_2_-2 to C-1 and C-3, from H_3_-19 to C-1, C-5, C-9, and C-10, from H_3_-21 to C-17, C-20, and C-22, from H_3_-18 to C-7, C-8, C-9, and C-14, and from H_3_-30 to C-8, C-13, C-14, and C-15. Key correlations from H-24 to C-20, and from H_3_-27 to C-24 and C-25 verified the 20,24-epoxy linkage and an additional hydroxyl group linked to C-25. Furthermore, a significant HMBC correlation between the anomeric hydrogen H-1′ and C-12 placed the glycoside moiety properly. Moreover, the pentose moiety was assigned as α-l-arabinopyranosyl based on the NMR data and comparison with those dammarane derivatives from this plant [14,20,21], as well as further acid hydrolysis and co-TLC with an authentic l-arabinose sample [22]. The relative configuration of **1** was determined by NOESY experiments (Figure 2). Strong NOESY correlations of H-5/H-9, H-9/H_3_-30, H-17/H_3_-30, and H-12/H-17 indicated that H-5, H-9, H-12, H-17, and H_3_-30 are α-oriented. The β-orientation of H-13, H_3_-18, and H_3_-19 were then verified by NOESY correlations of H-13/H_3_-18 and H_3_-18/H_3_-19. Because free rotation of the C-17–C-20 single bond was restricted to some extent in these triterpenoids with a 20,24-epoxyside chain [14,20], the configurations at C-20 and C-24 were then determined to be 20*S* and 24*R* based on key NOESY correlations of H-17/H_3_-21 and H_3_-21/H-24, respectively. As the absolute configuration on the main skeleton of these *seco*-dammarane triterpenoids have already been clarified by techniques such as single-crystal X-ray diffraction analysis with Cu K*α* irradiation [23], compound **1** was identified as (20*S*,24*R*)-20,24-epoxy-25-hydroxy-12β-(α-l-arabinopyranosyloxy)-3,4-*seco*-dammara-4(28)-en-3-oic acid.

Compound **2** exhibited a molecular formula of C_3__6_H_60_O_9_ based on its HRESIMS data. The NMR spectrum (Table 1) of **2** showed high similarities to that of **1** except for an additional methoxy group, as indicated by a ^1^H-NMR methyl singlet at δ_H_ 3.64 (3H, s, OCH_3_) and one ^13^C-NMR carbon signal at *δ*_C_ 52.1 (OCH_3_). Furthermore, an HMBC correlation between OCH_3_ and C-3 revealed that **2** was a 3-methyl ester derivative of **1**. The configuration of **2** was further confirmed the same as **1** by NOESY experiments. Ultimately, the structure of **2** was elucidated as (20*S*,24*R*)-20,24-epoxy-25-hydroxy-12β-(α-l-arabinopyranosyloxy)-3,4-*seco*-dammara-4(28)-en-3-oic acid methyl ester.

Compound **3** is a white amorphous powder. Its molecular formula was established as C_37_H_62_O_9_ by HRESIMS (*m*/*z* 673.4288 [M + Na]^+^, calcd for C_37_H_62_O_9_Na, 673.4292) and ^13^C-NMR data (Table 1). The 1D NMR data (Table 1) were almost identical to those of **2** except for the replacement of the arabinopyranosyl moiety in **2** by a quinovopyranosyl unit in **3**. The sugar linkage at C-12 was deduced by the HMBC correlation between H-1′ (δ_H_ 4.36) and C-12 (δ_C_ 76.5). The closely comparable NMR spectra for the sugar moiety of **3** to those of co-isolated known compounds **12**, **13**, **14**, and **15**, and the previously reported dammarane derivativesfrom this plant [14,20,21], together with an acid hydrolysis experiment and co-TLC with an authentic d-quinovose sample [22], suggested that the same β-d-quinovopyranosyl unit in **3**. The configuration of **3** was also confirmed by NOESY experiment. Therefore, compound **3** was characterized as (20*S*,24*R*)-20,24-epoxy-25-hydroxy-12β-(β-d-quinovopyranosyloxy)-3,4-*seco*-dammara-4(28)-en-3-oic acid methyl ester.

**Figure 3:**
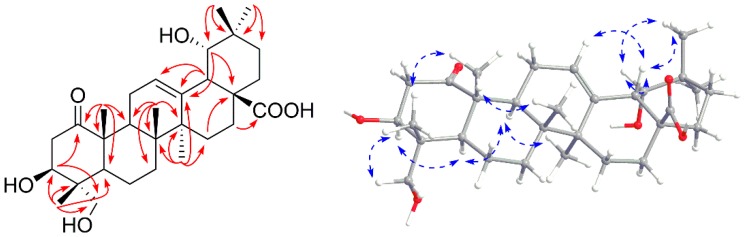
Key HMBC (red arrows) and NOESY (blue dotted double arrows) correlations of **4**.

Compound **4** was obtained as a white amorphous powder. It showed a molecular formula of C_30_H_46_O_6_ on the basis of the HRESIMS (*m*/*z* 525.3185 [M + Na]^+^, calcd for C_30_H_46_O_6_Na, 525.3192). The IR absorption bands at 3414, 2946 and 1695 cm^−1^ indicated the presence of hydroxyl groups, aliphatic C‒H and carbonyl groups, respectively. In the ^1^H-NMR spectrum (Table 1), six methyl singlets at δ_H_ 0.97 (s, H_3_-30), 0.94 (s, H_3_-29), 1.33 (s, H_3_-27), 0.85 (s, H_3_-26), 1.35 (s, H_3_-25), and 0.88 (s, H_3_-24), an oxygen-bearing methylene group at δ_H_ 3.33 (d, *J* = 11.2 Hz, H-23a) and 3.50 (d, *J* = 11.2 Hz, H-23b), and two oxygenated methine groups at δ_H_ 3.82 (dd, *J* = 12.1, 4.9 Hz, H-3) and 3.27 (d, *J* = 3.6 Hz, H-19) showed a pentacyclic triterpenoid structure. The ^13^C-NMR (Table 1) and DEPT (135) spectra revealed a carboxyl group at δ_C_ 182.9, a keto carbonyl group at δ_C_ 215.4, and two olefinic carbons at δ_C_ 125.3 and 144.0, as well as three oxygen-bearing carbons at δ_C_ 73.5 (C-3), 82.4 (C-19), and 65.9 (C-23). Thus, compound **4** was assigned as an olean-type triterpenoid. The NMR data of **4** were closely related to those of the known compound, 3β,23-dihydroxy-1-oxo-olean-12-en-28-oic acid [24], except for an additional hydroxyl group. 2D NMR experiments further confirmed the structure of **4**. In the HMBC spectrum (Figure 3), the olean-type skeleton was confirmed mainly by correlations from H_3_-29 and H_3_-30 to C-19, C-20, and C-21, from H_3_-27 to C-8, C-13, C-14, and C-15, from H_3_-26 to C-7, C-8, C-9, and C-14, from H_3_-25 to C-1, C-5, C-9, and C-10, from H_3_-24 to C-3, C-4, C-5, and C-23, and from H-18 to C-12 and C-13. The hydroxyl group at C-19 was verified by the HMBC correlations from H-19 (δ_H_ 3.27) to C-13 (δ_C_ 144.0), and C-17 (δ_C_ 47.0). The configuration was determined by NOESY correlations of H-3/H-23b, H-3/H-5, H-5/H-9, and H-9/H_3_-27 for the α-oriented hydrogens. Meanwhile, the key β-oriented hydrogens were identified by NOESY correlations of H_3_-24/H_3_-25, H_3_-25/H_3_-26, H-12/H-18, and H-18/H_3_-30 (Figure 3). The configuration of the hydroxyl group at C-19 was deduced to be α-oriented on the basis of observed NOE correlations between H-18 and H-19, and between H-19 and H_3_-30. Accordingly, compound **4** was deduced as 3β,19α,23-trihydroxy-1-oxo-olean-12-en-28-oic acid.

By comparing their NMR data with those reported in the literatures, the eleven known compounds (Table S1) were identified asarjunolic acid (**5**) [25], cyclocaric acid B (**6**) [26], 1α, 3β-dihydroxy-olean-12-en-28-oic acid (**7**) [27], punicaone (**8**) [28], olean-12-en-1β,3β,28-triol (**9**) [29], ursolic acid (**10**) [30], asiatic acid (**11**) [31], cyclocarioside K (**12**) [20], cyclocarioside H (**13**) [14], cyclocariosideI (**14**) [32], and cyclocarioside B (**15**) [33], respectively.

### 2.2. Glucose Uptake Assay

**Figure 4:**
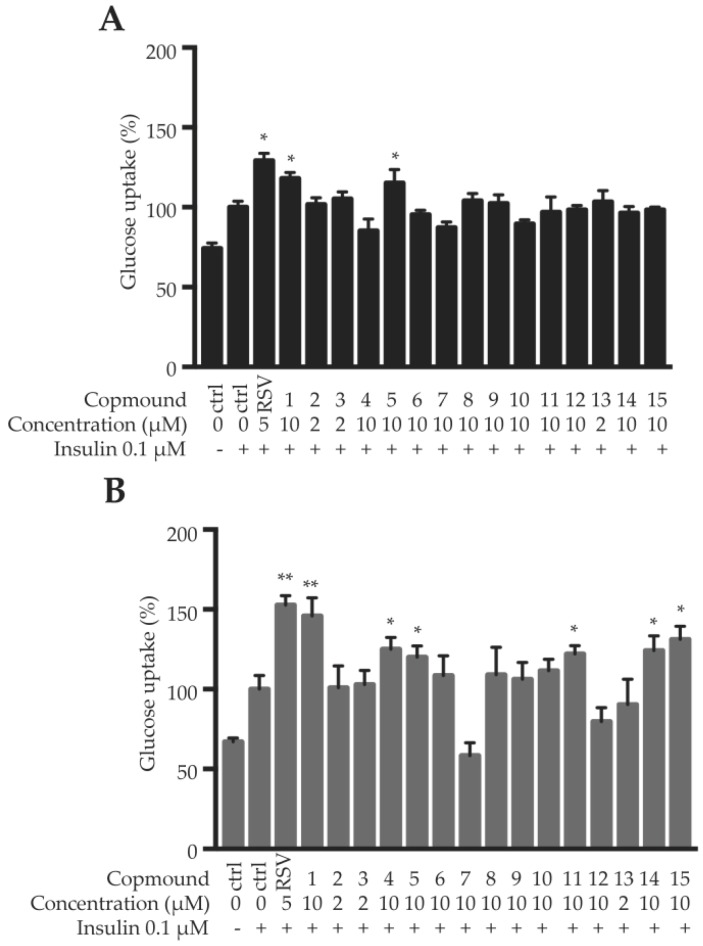
Glucose uptake of the compounds in C2C12 myotubes (**A**) and 3T3-L1 adipocytes (**B**). Data are shown as mean ± SD, *n* = 6. * *p* < 0.05, ** *p* < 0.01, compound vs. insulin.

Herein, the fully differentiated 3T3-L1 adipocytes and C2C12 myotubes were used to evaluate the insulin sensitizing effects of the compounds **1**–**15**, using 2-(*N*-(7-nitrobenz-2-oxa-1,3-diazol-4-yl) amino)-2-deoxyglucose (2-NBDG) uptake assay. Firstly, cell viability was determined using 3-(4,5-dimethylthiazol-2-yl)-2,5-diphenyltetrazolium bromide (MTT) assay. The cell viability above 90% were considered as non-cytotoxicity. As shown in Table S2, the maximum safe dosages for most compounds were 10 μM, except compounds **2**, **3** and **13** (2 μM), in both 3T3-L1 adipocytes and C2C12 myotubes. Under the maximum safe dosages, compounds **1** and **5** significantly enhanced insulin-stimulated glucose uptake in C2C12 myotubes; and compounds **1**, **4**, **5**, **11**, **14** and **15** remarkably promoted insulin-stimulated glucose uptake in 3T3-L1 adipocytes (Figure 4). Among them, compound **1** showed the most potent insulin sensitizing effect, increasing around 18% and 46% glucose uptake in C2C12 myotubes and 3T3-L1 adipocytes, respectively. Resveratrol (RSV, 5 μM) was used as a positive control, which increased about 29% and 53% glucose uptake in C2C12 myotubes and 3T3-L1 adipocytes, respectively (Figure 4). The existence of 3-hydroxyl and 23-hydroxymethyl groups is important for the activities of the pentacyclic triterpenoids.

### 2.3. Compound ***1*** Enhances Insulin Sensitivity in 3T3-L1 Adipocytes through Activating AMP-Activated Protein Kinase (AMPK)-p38 Pathway

**Figure 5:**
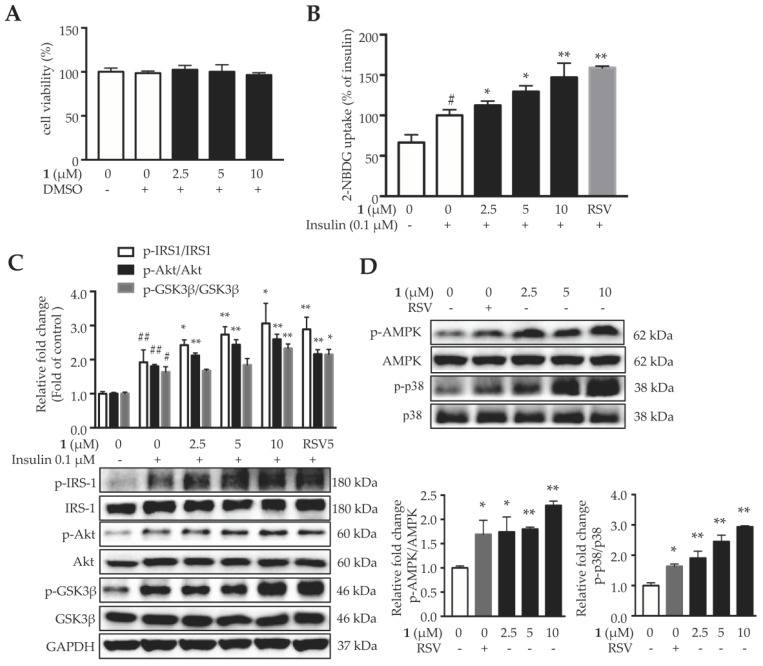
Compound **1** enhances insulin sensitivity in 3T3-L1 adipocytes through activating AMPK-p38 pathway. (**A**) Cell viability of mature 3T3-L1 adipocytes when treated with different concentrations of compound **1** for 24 h. (**B**) Compound **1** promotes insulin-stimulated glucose uptake in 3T3-L1 adipocytes. (**C**) Compound **1** activates the proteins in insulin signaling pathway, including IRS-1, Akt and GSK-3β. (**D**) Compound **1** increases the phosphorylation of AMPK and p38MAPK. Data are shown as mean ± SD, *n* = 6. ^#^ *p* < 0.05, ^##^ *p* < 0.01, vehicle vs. insulin. * *p* < 0.05, ** *p* < 0.01, compound **1** vs. DMSO.

Compound **1** didn’t show obvious cytotoxicity in 3T3-L1 adipocytes up to 10 μM (Figure 5A). As shown in Figure 5B, compound **1** increased insulin-stimulated glucose uptake in 3T3-L1 adipocytes in a dose-dependent manner. Next, the key proteins in the insulin signaling pathway were analyzed. As shown in Figure 5C, insulin increased the phosphorylation of IRS-1, Akt, and glycogen synthase kinase 3β (GSK-3β), and compound **1** further enhanced the phosphorylation of these proteins. These results indicated that compound **1** enhances insulin sensitivity and promotes glucose uptake by activating the insulin signaling pathway.

Previous reports have revealed that triterpenoids possess hypoglycemic effects through modulating insulin sensitivity in adipose, muscle or liver, and/or insulin secretion in pancreas. Ursolic acid (**10**), a pentacyclic triterpenoid from many different plants, promotes glucose uptake in adipocytes through enhancing PI3K level and glucose transporter 4 (GLUT4) translocation [7]. Corosolic acid, which is rich in Banaba (*Lagerstroemia speciosa*) leaf, actives insulin signaling pathway via transmitting the signals to PI3K/Akt as well as extracellular regulated protein kinases (ERK) pathways [34,35]. Moreover, eburicoic acid, a triterpenoid from *Antrodia camphorata*, displays antidiabetic and hypolipidemic properties in HFD-fed mice through activating AMPK [36].

AMPK, the energy-sensing enzyme, plays a central role in regulating glucose metabolism, which is positively correlated with insulin sensitivity in different tissues [37,38]. P38 mitogen-activated protein kinase (MAPK) exhibits wide-spectrum roles in controlling energy metabolism of adipose tissues [39]. P38 is a downstream component of the AMPK signaling pathway and is essential for insulin stimulated glucose uptake in adipocytes [40]. Several natural compounds are able to increase glucose uptake and improve hyperglycemia through activating AMPK-p38 pathway, including capsaicin [41], curcumin [42] and berberine [43]. Compound **1** increased the phosphorylation of AMPK and its downstream signal p38 in 3T3-L1 adipocytes, in a dose-dependent manner (Figure 5D). These results suggested that compound **1** might activate AMPK and p38 MAPK, to enhance insulin-stimulated glucose uptake in adipocytes.

Most triterpenoids are difficult to permeate through cell membranes. However, chronic intake of triterpene-rich extracts increases their bioavailability and accumulation in circulation and tissues [44]. Cyclodextrins was also reported to improve the bioavailability of triterpenoids [45]. The poor bioavailability of triterpenoids seriously affected their clinical efficacies and limited their applications [46]. To elicit their desired pharmacological effect, enzymatic or chemical transformation can be applied [47]. The carriers such as poly-drug conjugates, micelles, nanoparticles, and liposomes might be used to improve drug delivery efficiency [48,49].

## 3. Materials and Methods

### 3.1. General Experimental Procedures

Optical rotations were acquired in MeOH using a p-1010 polarimeter (Jasco, Easton, MD, USA). Infrared spectra were measured on an Avatar 370 FT-IR spectrometer (Nicolet, Carlsbad, CA, USA) with KBr disks. HRESIMS were acquired on a LCQ^DECA^ XP instrument (Finnigan, San Jose, CA, USA) and a Q-TOF 1290 LC/6224 MS system (Agilent, Santa Clara, CA, USA). NMR spectra were recorded on an AVANCE III 500 MHz spectrometer (Bruker, Zürich, Switzerland) with tetramethylsilane(TMS) as the internal standard. Silica gel (200–300 mesh, Qingdao Haiyang Chemical Co., Ltd., Qingdao, China), MCI gel (75–150 μm, Mitsubishi Chemical Industries Ltd., Tokyo, Japan), D-101 macroporous resin (Chemical Plant of Nankai University, Tianjin, People’s Republic of China), C_18_ reverse-phased silica gel (40–75 μm, Fuji, Kasugai, Japan), and Sephadex LH-20 gel (Amersham Pharmacia Biotech., Piscataway, NJ, USA) were performed on column chromatography. Precoated silica gel GF254 plates (Qingdao Haiyang Chemical Co., Ltd., Qingdao, China) were used for TLC. D-Quinovose and L-arabinose were purchased from Nantong Feiyu Biological Technology Co., Ltd. (Nantong, Jiangsu, China). All solvents were of analytical grade (Tianjin Yongda Chemical Reagent Co., Ltd., Tianjin, China).

### 3.2. Plant Material

The leaves of *Cyclocarya paliurus* (Batalin) Iljinsk. (Juglandaceae) were collected in Wencheng County, Wenzhou City, Zhejiang Province, China, in April 2016, and authenticated by one of the authors Jian-Xia Mo. A voucher specimen (accession number CP-2016-I) was deposited in the Institute of Modern Chinese Medicine, Zhejiang University (Hangzhou, China).

### 3.3. Extraction and Isolation

The air-dried leaves of *C. paliurus* (5 kg) were powdered and extracted with 70% EtOH (3 × 25 L) at room temperature. A crude extract (831.0 g) was obtained after removal of the solvent under reduced pressure. The extract was then suspended in H_2_O (2 L) and successively partitioned with petroleum ether, EtOAc, and *n*-BuOH(volume ratio of 1:1) to give three fractions, CPP (129.9 g), CPE (227.2 g), and CPB (136.2 g), respectively. The EtOAc fraction CPE was first subjected to a D-101 macroporous resin column eluted with aqueous EtOH (30% to 95%, stepwise) to afford seven subfractions of CPEA–CPEG. CPEC (4.0 g) was separated on a Sephadex LH-20 column (CH_2_Cl_2_‒MeOH, 1:1) to yield three subfractions (CPEC1–CPEC3). CPEC2 (2.3 g) was further chromatographed over a C_18_ reversed-phase column (from 30% to 100% aqueous MeOH), followed by a silica gel column (CH_2_Cl_2_‒MeOH, 40:1) to obtain compound **4** (7.0 mg). CPED (37.9 g) was purified by an MCI gel column eluted with aqueous MeOH (30% to 100%, stepwise) to give seven subfractions of CPED1–CPED7. CPED2 (5.5 g) was further separated by a C_18_ reversed-phase silica gel column (from 30% to 100% aqueous MeOH) to afford five subfractions of CPED2a‒CPED2e. CPED2b (1.2 g) was further chromatographed on a silica gel column (CH_2_Cl_2_‒MeOH, 30:1 to 5:1) to afford compounds **13** (33.5 mg) and **14** (51.3 mg). CPED2d (0.7 g) was subjected to a silica gel column (CH_2_Cl_2_‒MeOH, 20:1 to 0:1) to give compound **12** (42.2 mg). Next, CPED3 (6.9 g) was fractionated by a silica gel column (CH_2_Cl_2_–acetone, 8:1 to 0:1) and purified by repeated recrystallization to yield compounds **6** (1.6 g) and **5** (1.2 g). Compound **11** (50.4 mg) was obtained from CPED5 (0.8 g) via a Sephadex LH-20 column (CH_2_Cl_2_‒MeOH, 1:1). CPEE (30.3 g) was subjected to an MCI gel column eluted with aqueous MeOH (30% to 100%, stepwise) to give eight subfractions of CPEE1–CPEE8. CPEE4 (5.3 g) was performed on a C_18_ reversed-phase silica gel column (from 30% to 100% aqueous MeOH) to give five subfractions (CPEE4a–CPEE4e). Compound **15** (29.0 mg) was obtained from CPEE4a (0.9 g) by a silica gel column (EtOAc‒MeOH, 50:1 to 18:1), and compound **1** (22.0 mg) was from CPEE4b (1.1 g). CPEE4d (1.8 g) was further chromatographed on a silica gel column (CH_2_Cl_2_‒MeOH, 45:1 to 18:1) to yield compounds **3** (21.4 mg) and **2** (23.9 mg). CPEE6 (2.3 g) was passed through a silica gel column (CH_2_Cl_2_‒MeOH, 60:1 to 0:1) to give compounds **8** (25.8 mg) and **9** (15.5 mg). CPEF (8.4 g) was separated on a silica gel column (petroleum ether–acetone, 15:1 to 0:1) to give seven subfractions of CPEF1–CPEF7. CPEF4 (0.9 g) was purified by a Sephadex LH-20 column (CH_2_Cl_2_‒MeOH, 1:1) to afford compound **10** (132.0 mg). CPEF6 (0.4 g) was further chromatographed on a silica gel column (CH_2_Cl_2_‒EtOAc, 16:1 to 6:1) to obtain compound **7** (35.8 mg).

*(20S,24R)-20,24-Epoxy-25-hydroxy-12β-(α-l-arabinopyranosyloxy)-3,4-seco-dammara-4(28)-en-3-oic acid* (**1**): White amorphous powder; [α${]}_{D}^{25}$+ 27.2 (*c* 0.09, MeOH); IR (KBr) *ν*_max_ 3405, 2964, 1699, 1685, 1455, 1394, 1087, 1002, 946 cm^−1^; ^1^H- and ^13^C-NMR data, see Table 1; ESIMS (positive) *m*/*z* 645 [M + Na]^+^; ESIMS (negative) *m*/*z* 621 [M − H]^−^; HRESIMS (positive) *m*/*z* 645.3978 [M + Na]^+^ (calcd for C_35_H_58_O_9_Na, 645.3979).

*(20S,24R)-20,24-Epoxy-25-hydroxy-12β-(α-l-arabinopyranosyloxy)-3,4-seco-dammara-4(28)-en-3-oic acid methyl ester* (**2**): White amorphous powder; [α${]}_{D}^{25}$+ 24.9 (*c* 0.10, MeOH); IR (KBr) *ν*_max_ 3418, 2963, 1721, 1634, 1454, 1377, 1087, 1001, 946, 893 cm^−1^; ^1^H- and ^13^C-NMR data, see Table 1; ESIMS (positive) *m*/*z* 637 [M + H]^+^; ESIMS (negative) *m*/*z* 681 [M + HCOO]^−^; HRESIMS (positive) *m*/*z* 659.4127 [M + Na]^+^ (calcd for C_36_H_60_O_9_Na, 659.4135).

*(20S,24R)-20,24-Epoxy-25-hydroxy-12β-(β-d-quinovopyranosyloxy)-3,4-seco-dammara-4(28)-en-3-oic acid methyl ester* (**3**): White amorphous powder; [α${]}_{D}^{25}$+ 17.5 (*c* 0.08, MeOH); IR (KBr) *ν*_max_ 3416, 2965, 1715, 1635, 1455, 1394, 1166, 1067, 893 cm^−1^; ^1^H- and ^13^C-NMR data, see Table 1; ESIMS (positive) *m*/*z* 651 [M + H]^+^; ESIMS (negative) *m*/*z* 695 [M + HCOO]^−^; HRESIMS (positive) *m*/*z* 673.4288 [M + Na]^+^ (calcd for C_37_H_62_O_9_Na, 673.4292).

*3β,19α,23-Trihydroxy-1-oxo-olean-12-en-28-oic acid* (**4**): White amorphous powder; [α${]}_{D}^{25}$+ 62.2 (*c* 0.10, MeOH); IR (KBr) *ν*_max_ 3414, 2946, 1695, 1463, 1385, 1040, 955 cm^−1^; ^1^H- and ^13^C-NMR data, see Table 1; ESIMS (positive) *m*/*z* 525 [M + Na]^+^; ESIMS (negative) *m*/*z* 537 [M + Cl]^−^; HRESIMS (positive) *m*/*z* 525.3185 [M + Na]^+^ (calcd for C_30_H_46_O_6_Na, 525.3192).

### 3.4. Acid Hydrolysis of Compounds ***1***–***3***

The configuration of sugar moieties was established according to the published method with some modifications [22,50]. Compounds **1**–**3** (5.0 mg of each)were refluxed with 10% HCl in EtOH (10 mL) for 6 h, respectively. Each reaction mixture was diluted with H_2_O and extracted with EtOAc (3 × 15 mL). After neutralizing with Na_2_CO_3_, the presence of L-arabinose inthe aqueous layer for **1**, **2** was detected by co-TLC (CH_2_Cl_2_-MeOH-H_2_O, 12:8:1, *R_f_* = 0.65) with an authentic sample, and that of D-quinovose for **3** was also confirmed by co-TLC (CH_2_Cl_2_-MeOH-H_2_O, 12:8:1, *R_f_* = 0.55) with an authentic sample.

### 3.5. Reagents

All the isolates and RSV were dissolved in DMSO (less than 0.1% in the cell-based assays). Fetal bovine serum (FBS), calf serum (CS), horse serum (HS), penicillin-streptomycin (P/S), Dulbecco’s modified Eagle’s medium (DMEM), and phosphate-buffered saline (PBS) powder were purchased from Life Technologies (Grand Island, NY, USA). All the chemicals (analytical grade) were obtained from Sigma-Aldrich (St. Louis, MO, USA), except those otherwise specified. Antibody details were as follows: p-p38 (sc-166182), p38 (sc-81621), Akt (sc-8312), p-Akt (sc-7985), AMPK (sc-25792), p-AMPK (sc-33524), p-IRS1 (sc-17196) and GAPDH (sc-25778) were purchased from Santa Cruz Biotechnology, Inc. (Santa Cruz, CA, USA). p-GSK3β (#9323), GSK3β (#12456) and IRS1 (#2382) were from Cell Signaling (Danvers, MA, USA).

### 3.6. Cell Culture and Treatment

Mouse C2C12 myoblasts and 3T3-L1 preadipocytes were obtained from ATCC (Manassas, VA, USA). C2C12 cells were cultured in DMEM supplied with 10% FBS and 1% P/S. After 70‒80% confluence, the C2C12 cells were incubated with DMEM containing 1% P/S and 2% heat-inactivated HS for 4 days, to induce differentiation. Medium was changed every other day. The fully differentiated cells were treated with the different concentration of compounds for 24 h.

3T3-L1 preadipocytes were cultured in DMEM supplied with 10% CS and 1% P/S.2 Days post confluence, the cells were incubated with DMEM supplied with 10% FBS and DMI (1 μM dexamethasone, 0.5 mM 3-isobutyl-1-methylxanthine, and 5 μg/mL insulin) for 2 days. Subsequently, cells were maintained in DMEM supplied with 10% FBS and 5 μg/mL insulin for 6 days, and medium was refreshed every other day. On day 8, fully differentiated adipocytes were treated with compounds at indicated concentrations for 24 h.

### 3.7. Cell Viability Assay

Cell viability was determined by the 3-(4,5-dimethylthiazol-2-yl)-2,5-diphenyltetrazolium bromide (MTT) assay (Sigma-Aldrich) as described previously [51]. Briefly, cells were seeded in 96-well plates at a density of 5 × 10^4^ cells/well. 3T3-L1 adipocytes and C2C12 myotubes were treated with compounds at indicated concentrations for 24 h, respectively. Subsequently, cell viability was determined by incubation with 1 mg/mL MTT solution in DMEM at 37 °C for 4 h, followed by dissolving the violet-formazan crystals with 100 µL DMSO. The absorbance at 570 nm was measured by a SpectraMax M5 microplate reader (Molecular Devices, San Jose, CA, USA). Cell viability of each group was expressed as a percentage of the control group treated with DMSO.

### 3.8. Glucose Uptake Assay

Glucose uptake assay was followed as previous study [52]. Fully differentiated 3T3-L1 adipocytes and C2C12 myotubes were treated with compounds at indicated concentrations for 24 h. After washed with Krebs-Ringer’s phosphate (KRP) buffer (20 mM HEPES, 137 mM NaCl, 4.7 mM KCl, 1.2 mM MgSO_4_, 1.2 mM KH_2_PO_4_, 2.5 mM CaCl_2_, and 2 mM pyruvate; pH 7.4), cells were incubated in KRP buffer with 0.2% bovine serum albumin for 3 h. Next, cells were incubated with KRP buffer with 0.1 μM insulin for another 30 min, to stimulate glucose uptake. Cells were incubated for another 30 min in KRP containing 100 μM 2-NBDG after washed with KRP buffer once. The intracellular content of 2-NBDG was determined at an excitation wavelength of 475 nm and an emission wavelength of 550 nm using a SpectraMax M5 microplate reader. Finally, glucose uptake was normalized with protein content.

### 3.9. Western Blot Analysis

Western blot analysis was followed as previous study [53]. After washed twice with ice-cold PBS, 3T3-L1 adipocytes were lysed with cold RIPA buffercontaining freshly added phosphatase inhibitor cocktails and phenylmethylsulfonyl fluoride (PMSF), by incubating on ice for 30 min. The supernatants were collected after centrifuging the cell lysates at 17,400×*g* for 20 min at 4°C. BCA protein assay kit (Life Technologies, Grand Island, NY, USA) was used to quantified the protein concentration of each sample. Equal amount of proteins (30 μg) were separated by 8–10% sodium dodecyl sulfate polyacrylamide gel electrophoresis (SDS-PAGE) and transferred to polyvinylidene difluoride (PVDF) membranes (Bio-Rad Laboratories, Inc., Hercules, CA, USA). The membranes were blocked with 5% nonfat milk in TBST buffer (100 mM NaCl, 10 mM Tris-HCl, pH 7.5 and 0.1% Tween-20) for 1 h at room temperature and then incubated with specific primary antibodies in TBST buffer overnight at 4 °C. After washing with TBST buffer thrice, the membranes were incubated with a horseradish peroxidase conjugated secondary antibody for 2 h at room temperature. The specific signals were developed using a SuperSignal West Femto Maximum Sensitivity Substrate kit (Thermo, Rockford, IL, USA), and visualized using the ChemiDoc MP Imaging System.

### 3.10. Statistical Analysis

Statistical analyses were performed by Graphpad Prism 6 (GraphPad Software, San Diego, CA, USA). The data were expressed as mean ± S.D. based on at least three independent experiments. Statistical differences were accepted as significant at *p*-values less than 0.05, analyzing by one-way ANOVA with Dunnett’s test in multiple comparison.

## 4. Conclusions

**Figure 6:**
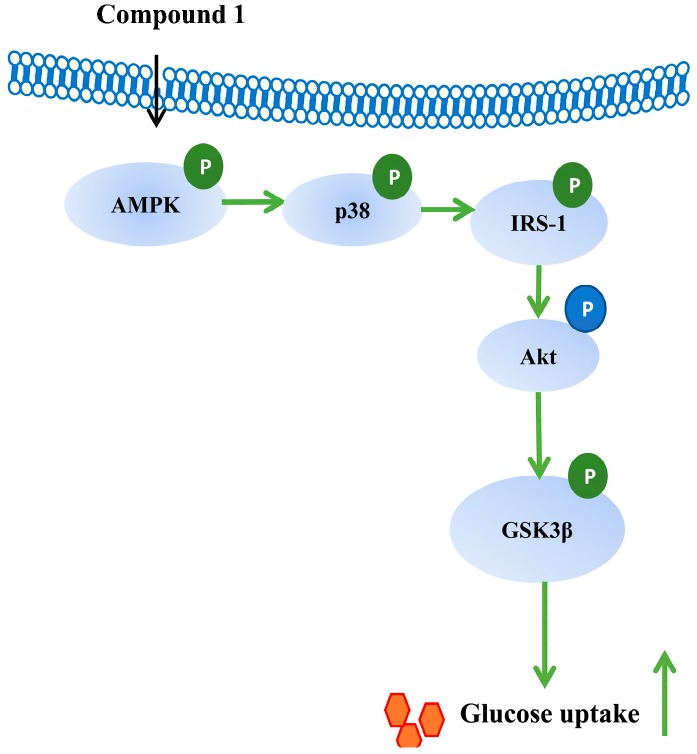
The mechanism of action of compound **1**.

Phytochemical investigation on the leaves of *C. paliurus* led to the isolation of 15 triterpenoids including three undescribed *seco*-dammarane triterpenoid glycosides **1**–**3**, one undescribed pentacyclic triterpenic acid **4**, and eleven known analogues. Among them, the three *seco*-dammarane triterpenoid glycosides **1**–**3** possessing 20,24-epoxy linkages are rarely found among the *seco*-dammarane triterpenoids from this genus. Several triterpenoids were found to enhance insulin-stimulated glucose uptake in both C2C12 myotubes and 3T3-L1 adipocytes. The insulin sensitizing effect of triterpenoids from *C. paliurus* was more potent in adipocytes than that in myotubes. Compound **1** enhances insulin sensitivity in adipocytes through activating AMPK-p38 pathway (Figure 6). Collectively, triterpenoids from *C. paliurus* could be developed as insulin sensitizers, which might have therapeutic potential for insulin resistance and hyperglycemia.

## Figures and Tables

**Table 1:** ^1^H-(500 MHz, *δ* in ppm, *J* in Hz) and ^13^C-NMR (125 MHz, *δ* in ppm) data of compounds **1**–**4** in CD_3_OD.

No.	1		2		3		4	
	δ_H_	δ_C_	δ_H_	δ_C_	δ_H_	δ_C_	δ_H_	δ_C_
1	a 1.53, m	38.4	a 1.47, m	38.3	a 1.42, m	38.0		215.4
	b 2.52, m		b 2.53, dt (13.3, 5.0)		b 2.46, m			
2	a 2.26, m	30.8	a 2.29, dt (13.3, 3.5)	30.7	a 2.27, dt (13.3, 3.3)	30.5	α 2.29, dd (11.9, 4.9)	44.8
	b 2.66, dt (12.9, 5.2)		b 2.74, dt (13.0, 5.0)		b 2.74, dt (13.3, 5.1)		β 3.12, t (11.9)	
3		180.2		178.3		177.9	3.82, dd (12.1, 4.9)	73.5
4		149.3		149.3		149.3		44.1
5	2.05, dd (12.7, 2.8)	53.0	2.04, dd (12.8, 2.9)	53.1	2.04, dd (12.8, 2.9)	53.1	1.39, m	47.9
6	α 1.86, m	27.4	α 1.85, m	27.4	α 1.85, m	27.5	α 1.57, m	18.6
	β 1.45, m		β 1.49, m		β 1.47, m		β 1.57, m	
7	α 1.59, m	35.8	α 1.59, dt (13.0, 3.3)	35.8	α 1.59, dt (12.9, 3.3)	35.7	α 1.55, m	33.3
	β 1.22, m		β 1.21, m		β 1.22, m		β 1.29, m	
8		40.9		40.9		40.9		40.5
9	1.95, m	45.4	1.95, m	45.4	1.93, m	45.5	2.40, m	40.8
10		41.9		41.9		41.9		53.6
11	α 2.46, m	33.8	α 2.46, dt (12.5, 4.1)	34.0	α 2.42, m	34.3	α 2.44, m	26.5
	β 1.34, m		β 1.35, m		β 1.37, m		β 1.88, ddd (12.5, 11.3, 5.4)	
12	4.07, dt (10.7, 4.7)	76.5	4.05, dt (10.8, 4.9)	76.6	4.03, dt (10.8, 5.0)	76.5	5.30, m	125.3
13	1.70, m	41.9	1.70, m	41.9	1.70, m	41.8		144.0
14		51.4		51.4		51.4		42.8
15	α 1.49, m	32.3	α 1.44, m	32.3	α 1.45, m	32.4	α 1.00, m	29.6
	β 1.13, m		β 1.12, m		β 1.12, m		β 1.72, m	
16	α 1.99, m	27.1	α 1.98, m	27.1	α 1.98, m	27.0	α 2.25, m	28.7
	β 1.81, m		β 1.80, m		β 1.80, m		β 1.59. m	
17	1.92, m	50.2	1.93, m	50.2	1.92, m	50.1		47.0
18	1.06, s	16.9	1.05, s	16.9	1.05, s	16.8	3.06, br s	45.5
19	1.10, s	20.6	1.09, s	20.6	1.09, s	20.5	3.27, d (3.6)	82.4
20		87.8		87.8		87.9		36.1
21	1.16, s	25.5	1.16, s	25.5	1.16, s	25.2	α 1.64, m	29.6
							β 1.00, m	
22	α 1.73, m	35.0	α 1.74, m	35.0	α 1.73, m	34.8	α 1.62, m	34.0
	β 1.65, m		β 1.67, m		β 1.66, m		β 1.78, m	
23	α 1.89, m	26.1	α 1.89, m	26.1	α 1.89, m	26.1	a 3.33, d (11.2)	65.9
	β 1.31, m		β 1.31, m		β 1.32, m		b 3.50, d (11.2)	
24	3.79, m	84.9	3.79, m	84.9	3.79, dd (7.8, 6.6)	84.9	0.88, s	13.2
25		72.7		72.6		72.8	1.35, s	15.9
26	1.19, s	26.6	1.19, s	26.6	1.21, s	26.8	0.85, s	18.2
27	1.16, s	24.4	1.16, s	24.5	1.15, s	24.6	1.33, s	25.1
28	a 4.72, d (1.4)	114.1	a 4.71, d (1.6)	114.1	a 4.70, d (1.6)	114.0		182.9
	b 4.85, d (1.4)		b 4.84, d (1.6)		b 4.84, d (1.6)			
29	1.77, s	24.0	1.76, s	24.0	1.76, s	23.9	0.94, s	28.7
30	1.00, s	16.9	1.00, s	16.9	0.99, s	16.9	0.97, s	25.2
1′	4.29, d (7.1)	101.2	4.28, d (7.1)	101.4	4.36, d (7.7)	100.4		
2′	3.50, dd (9.6, 7.1)	72.8	3.50, dd (9.6, 7.1)	72.8	3.18, dd (9.3, 7.7)	75.3		
3′	3.46, dd (9.6, 3.3)	74.6	3.46, dd (9.6, 3.3)	74.6	3.29, m	77.8		
4′	3.77, m	70.4	3.77, m	70.3	3.00, t (9.1)	77.1		
5′	3.54, dd (12.7, 0.8)	67.8	3.54, dd (12.7, 1.1)	67.7	3.26, m	73.0		
	3.88, dd (12.7, 1.9)		3.88, dd (12.7, 1.9)					
6′					1.26, d (6.1)	18.1		
OCH_3_			3.64, s	52.1	3.63, s	52.0		

## Supplementary Materials

The following are available online. Figure S1–S40: 1D and 2D NMR, MS, and IR spectra of compounds **1**–**4**; Table S1: Structures of known compounds; Table S2: Cytotoxicity of the isolates in C2C12 myotubes and 3T3-L1 adipocytes.
