# Supplementary material for: Triterpenoids from Cyclocarya paliurus that Enhance Glucose Uptake in 3T3-L1 Adipocytes
Source: Molecules. 2019 Jan 6;24(1):187. doi: 10.3390/molecules24010187 (PMC6337507; doi:10.3390/molecules24010187)
Supplement: Supplementary file 1 [file molecules-24-00187-s001.pdf]

Supplementary data

for

Triterpenoids from *Cyclocarya paliurus* that Enhance Glucose Uptake in 3T3-L1 Adipocytes

Zhu-Jun Fang [1,†], Sheng-Nan Shen [2,†], Jia-Min Wang [1], Yong-Jiang Wu [1], Chang-Xin Zhou [1], Jian-Xia Mo [1], Li-Gen Lin [2,*] and Li-She Gan [1,3,*]

[1] College of Pharmaceutical Sciences, Zhejiang University, 866 Yuhangtang Road, Hangzhou 310058, People's Republic of China; 11519027@zju.edu.cn (Z.-J.F.); 21819075@zju.edu.cn (J.-M.W.); yjwu@zju.edu.cn (Y.-J.W.); zhoucx10@zju.edu.cn (C.-X.Z.); mojx@zju.edu.cn (J.-X.M.)

[2] State Key Laboratory of Quality Research in Chinese Medicine, Institute of Chinese Medical Sciences, University of Macau, Macao 999078, People's Republic of China; yb57518@um.edu.mo (S.-N.S.)

[3] Hangzhou Institute of Innovative Medicine, Zhejiang University, 291 Fucheng Road, Hangzhou 310018, People's Republic of China

* Correspondence: lsgan@zju.edu.cn (L.-S. Gan); ligenl@um.edu.mo (L.-G. Lin); Tel.: +86-571-8820-8457 (L.-S. Gan)

[†] These authors contributed equally to this work.

List of Content

Contents

Figure S1. ^{1}H NMR spectrum (500 MHz, Methanol-d_4) of compound **1**.

Figure S2. ^{13}C NMR spectrum (125 MHz, Methanol-d_4) of compound **1**.

Figure S3. ESI MS (negative mode) spectrum of compound **1**.

Figure S4. ESI MS (positive mode) spectrum of compound **1**.

Figure S5. HMQC spectrum (500 MHz, Methanol-d_4) of compound **1**.

Figure S6. HMBC spectrum (500 MHz, Methanol-d_4) of compound **1**.

Figure S7. NOESY spectrum (500 MHz, Methanol-d_4) of compound **1**.

Figure S8. ^{1}H-^{1}H COSY spectrum (500 MHz, Methanol-d_4) of compound **1**.

Figure S9. DEPT 135 spectrum (125 MHz, Methanol-d_4) of compound **1**.

Figure S10. IR (KBr disc) spectrum of compound **1**.

Figure S11. ^{1}H NMR spectrum (500 MHz, Methanol-d_4) of compound **2**.

Figure S12. ^{13}C NMR spectrum (125 MHz, Methanol-d_4) of compound **2**.

Figure S13. ESI MS (negative mode) spectrum of compound **2**.

Figure S14. ESI MS (positive mode) spectrum of compound **2**.

Figure S15. HMQC spectrum (500 MHz, Methanol-d_4) of compound **2**.

Figure S16. HMBC spectrum (500 MHz, Methanol-d_4) of compound **2**.

Figure S17. NOESY spectrum (500 MHz, Methanol-d_4) of compound **2**.

Figure S18. ^{1}H-^{1}H COSY spectrum (500 MHz, Methanol-d_4) of compound **2**.

Figure S19. DEPT 135 spectrum (125 MHz, Methanol-d_4) of compound **2**.

Figure S20. IR (KBr disc) spectrum of compound **2**.

Figure S21. ^{1}H NMR spectrum (500 MHz, Methanol-d_4) of compound **3**.

Figure S22. ^{13}C NMR spectrum (125 MHz, Methanol-d_4) of compound **3**.

Figure S23. ESI MS (negative mode) spectrum of compound **3**.

Figure S24. ESI MS (positive mode) spectrum of compound **3**.

Figure S25. HMQC spectrum (500 MHz, Methanol-d_4) of compound **3**.

Figure S26. HMBC spectrum (500 MHz, Methanol-d_4) of compound **3**.

Figure S27. NOESY spectrum (500 MHz, Methanol-d_4) of compound **3**.

Figure S28. ^{1}H-^{1}H COSY spectrum (500 MHz, Methanol-d_4) of compound **3**.

Figure S29. DEPT 135 spectrum (125 MHz, Methanol-d_4) of compound **3**.

Figure S30. IR (KBr disc) spectrum of compound **3**.

Figure S31. ^{1}H NMR spectrum (500 MHz, Methanol-d_4) of compound **4**.

Figure S32. ^{13}C NMR spectrum (125 MHz, Methanol-d_4) of compound **4**.

Figure S33. ESI MS (negative mode) spectrum of compound **4**.

Figure S34. ESI MS (positive mode) spectrum of compound **4**.

Figure S35. HMQC spectrum (500 MHz, Methanol-d_4) of compound **4**.

Figure S36. HMBC spectrum (500 MHz, Methanol-d_4) of compound **4**.

Figure S37. NOESY spectrum (500 MHz, Methanol-d_4) of compound **4**.

Figure S38. ^{1}H-^{1}H COSY spectrum (500 MHz, Methanol-d_4) of compound **4**.

Figure S39. DEPT 135 spectrum (125 MHz, Methanol-d_4) of compound **4**.

Figure S40. IR (KBr disc) spectrum of compound **4**.

Table S1. Structures of known compounds.

Table S2. Cytotoxicity of the isolates in C2C12 myotubes and 3T3-L1 adipocytes.

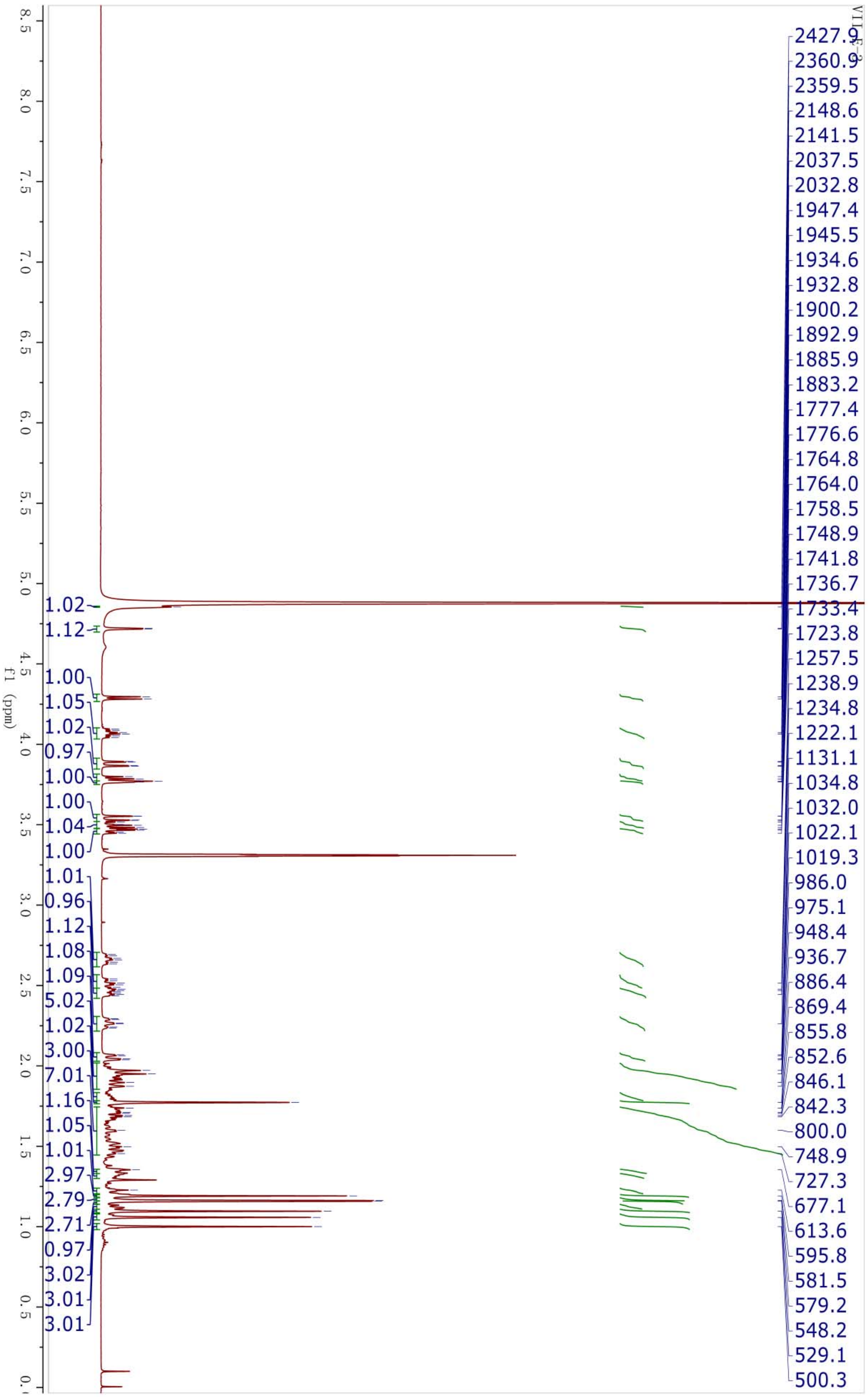
f1 (ppm)
8.5
8.0
7.5
7.0
6.5
6.0
5.5
5.0
4.5
4.0
3.5
3.0
2.5
2.0
1.5
1.0
0.5
1.02
1.12
1.00
1.05
1.02
0.97
1.00
1.00
1.04
1.00
1.01
0.96
1.12
1.08
1.09
5.02
1.02
3.00
7.01
1.16
1.05
1.01
2.97
2.79
2.71
0.97
3.02
3.01
3.01
2427.9
2360.9
2359.5
2148.6
2141.5
2037.5
2032.8
1947.4
1945.5
1934.6
1932.8
1900.2
1892.9
1885.9
1883.2
1777.4
1776.6
1764.8
1764.0
1758.5
1748.9
1741.8
1736.7
1733.4
1723.8
1257.5
1238.9
1234.8
1222.1
1131.1
1034.8
1032.0
1022.1
1019.3
986.0
975.1
948.4
936.7
886.4
869.4
855.8
852.6
846.1
842.3
800.0
748.9
727.3
677.1
613.6
595.8
581.5
579.2
548.2
529.1
500.3

Figure S1. ^{1}H NMR spectrum (500 MHz, Methanol-d_4) of compound **1**.

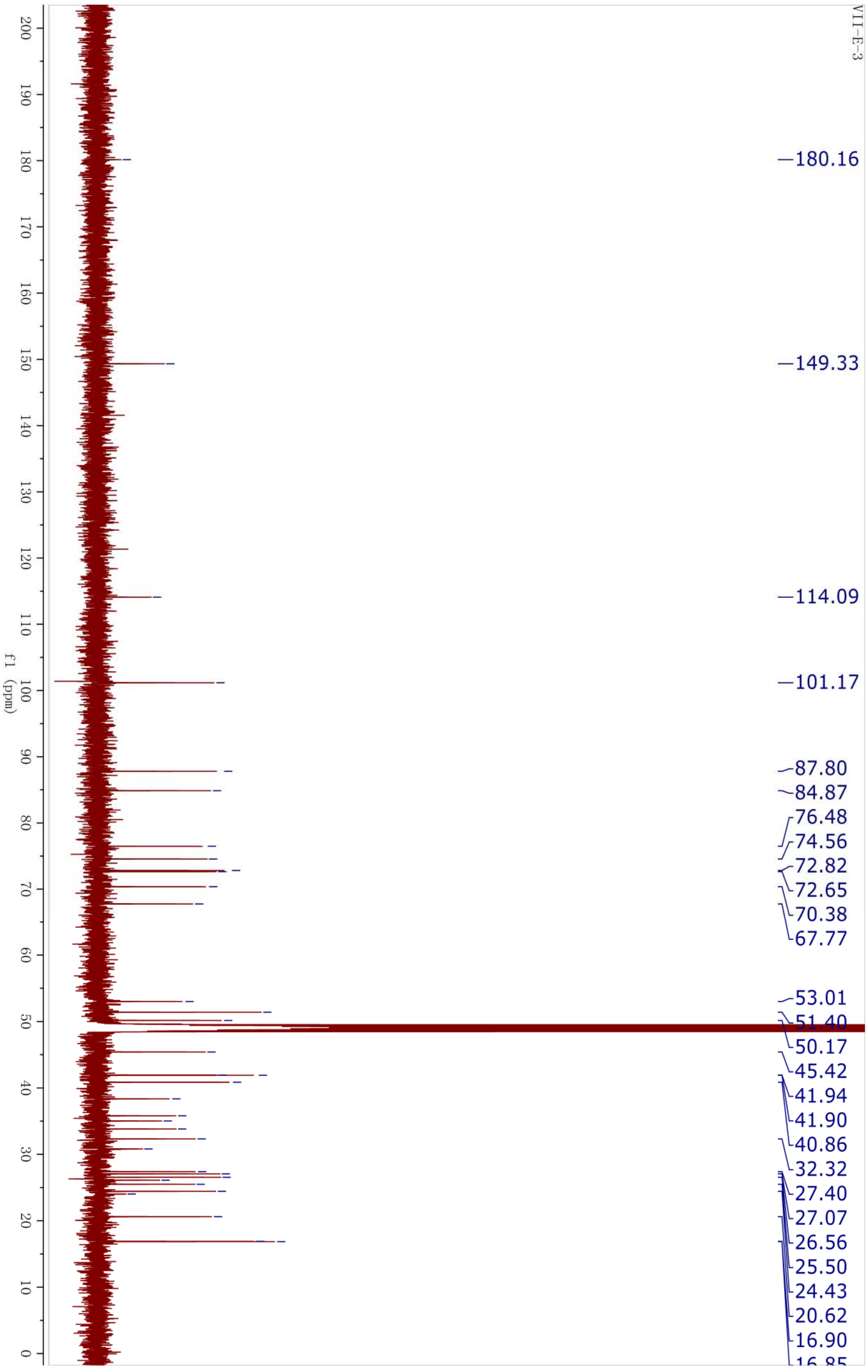

Figure S2. ^{13}C NMR spectrum (125 MHz, Methanol-d_4) of compound **1**.

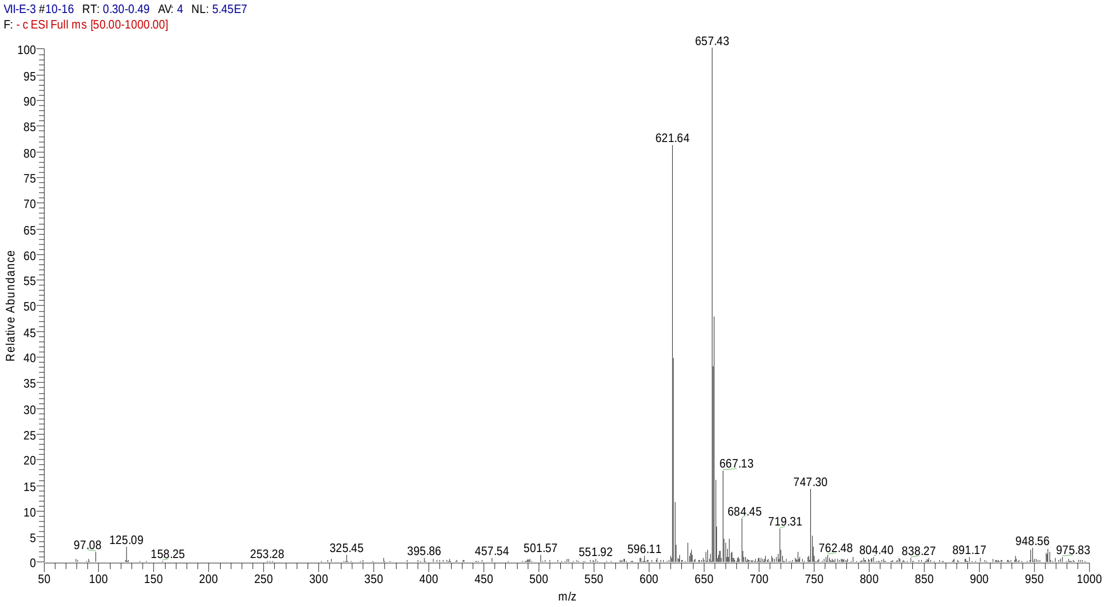

Figure S3. ESI MS (negative mode) spectrum of compound **1**.

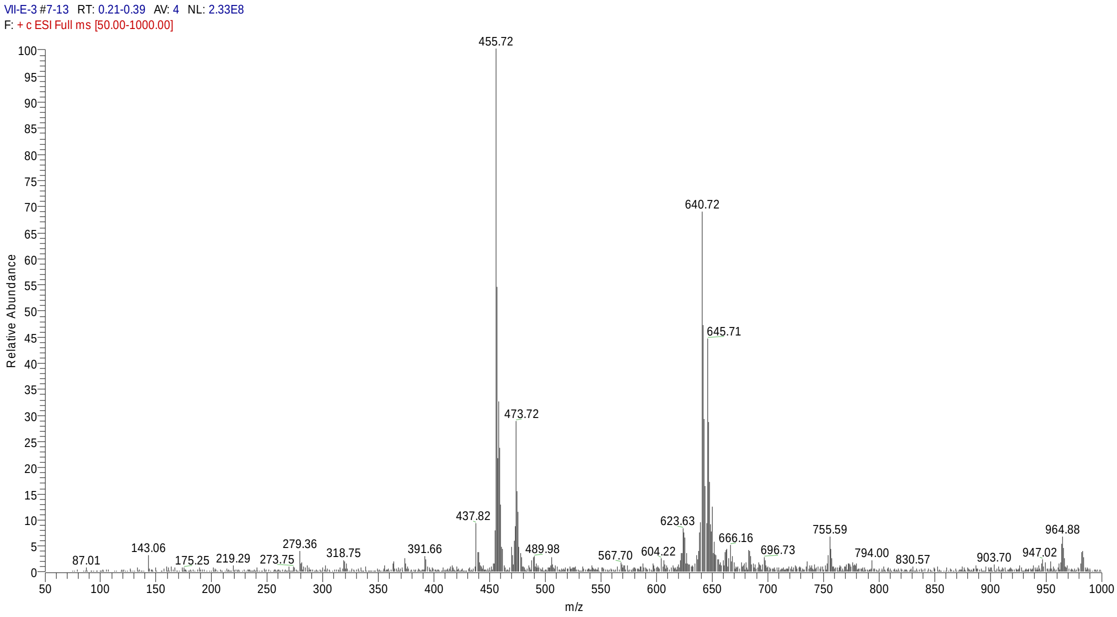

Figure S4. ESI MS (positive mode) spectrum of compound **1**.

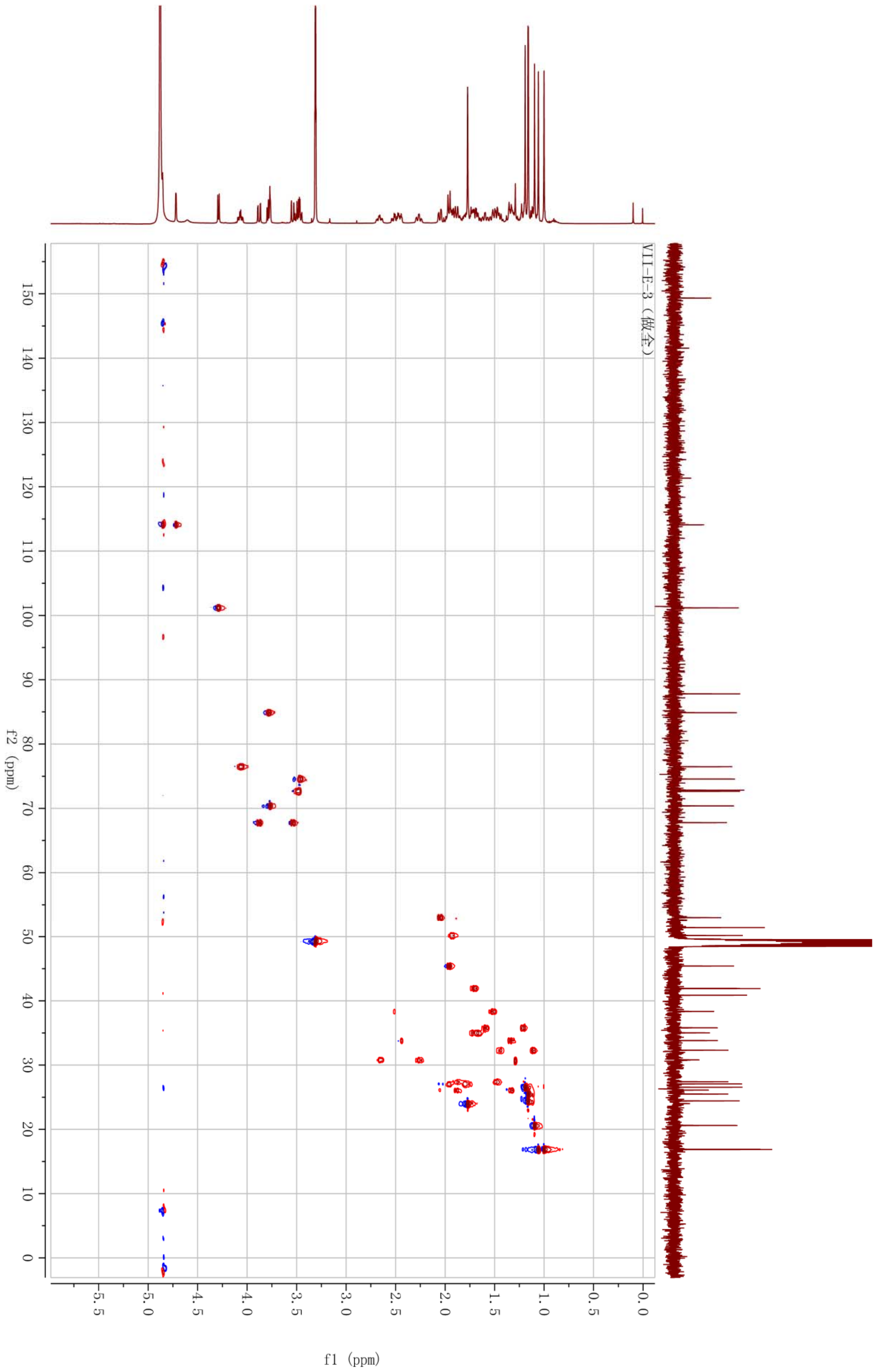

Figure S5. HMQC spectrum (500 MHz, Methanol-d_4) of compound **1**.

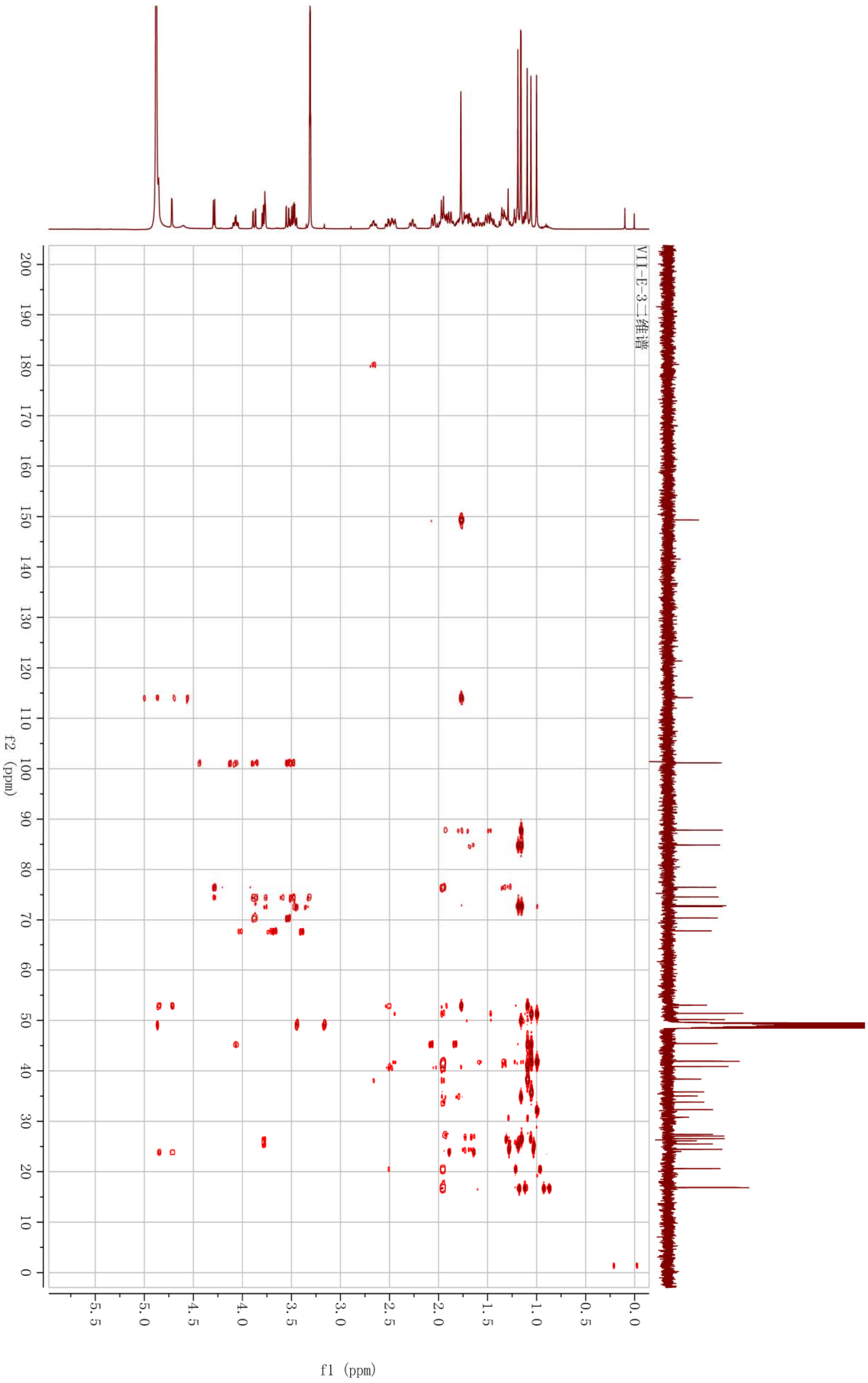

Figure S6. HMBC spectrum (500 MHz, Methanol-d_4) of compound **1**.

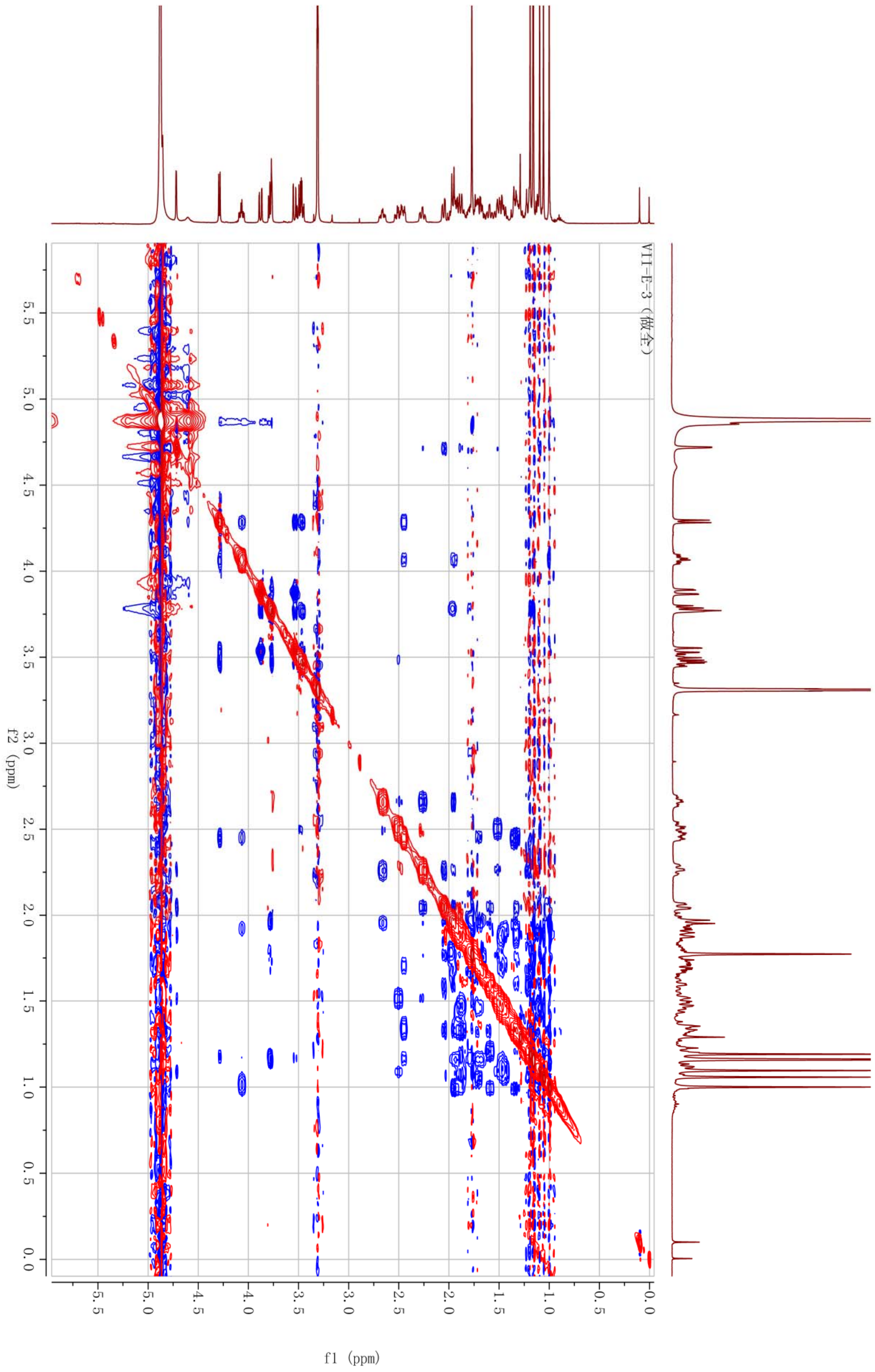

Figure S7. NOESY spectrum (500 MHz, Methanol-d_4) of compound **1**.

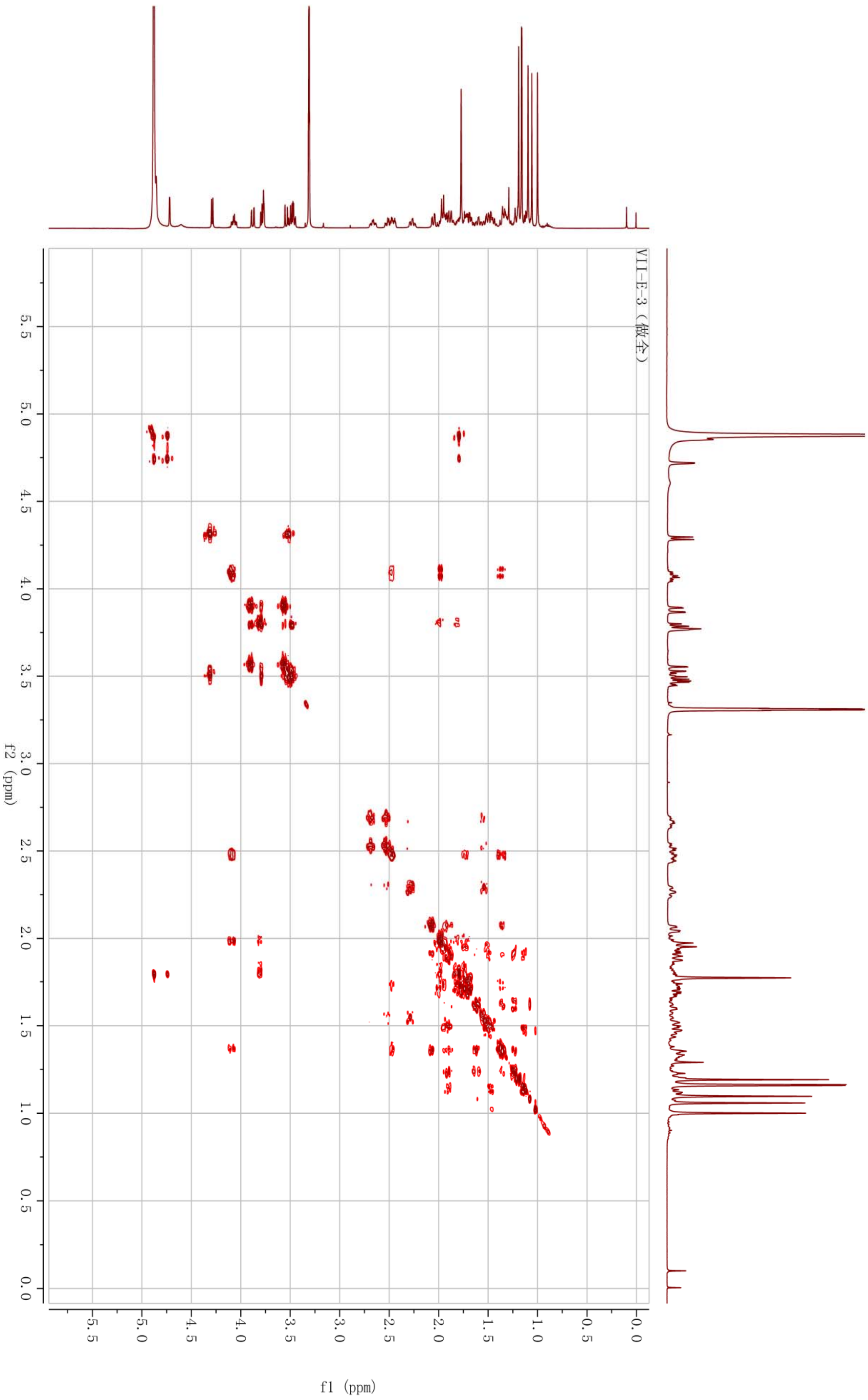

Figure S8. ^{1}H-^{1}H COSY spectrum (500 MHz, Methanol-d_4) of compound **1**.

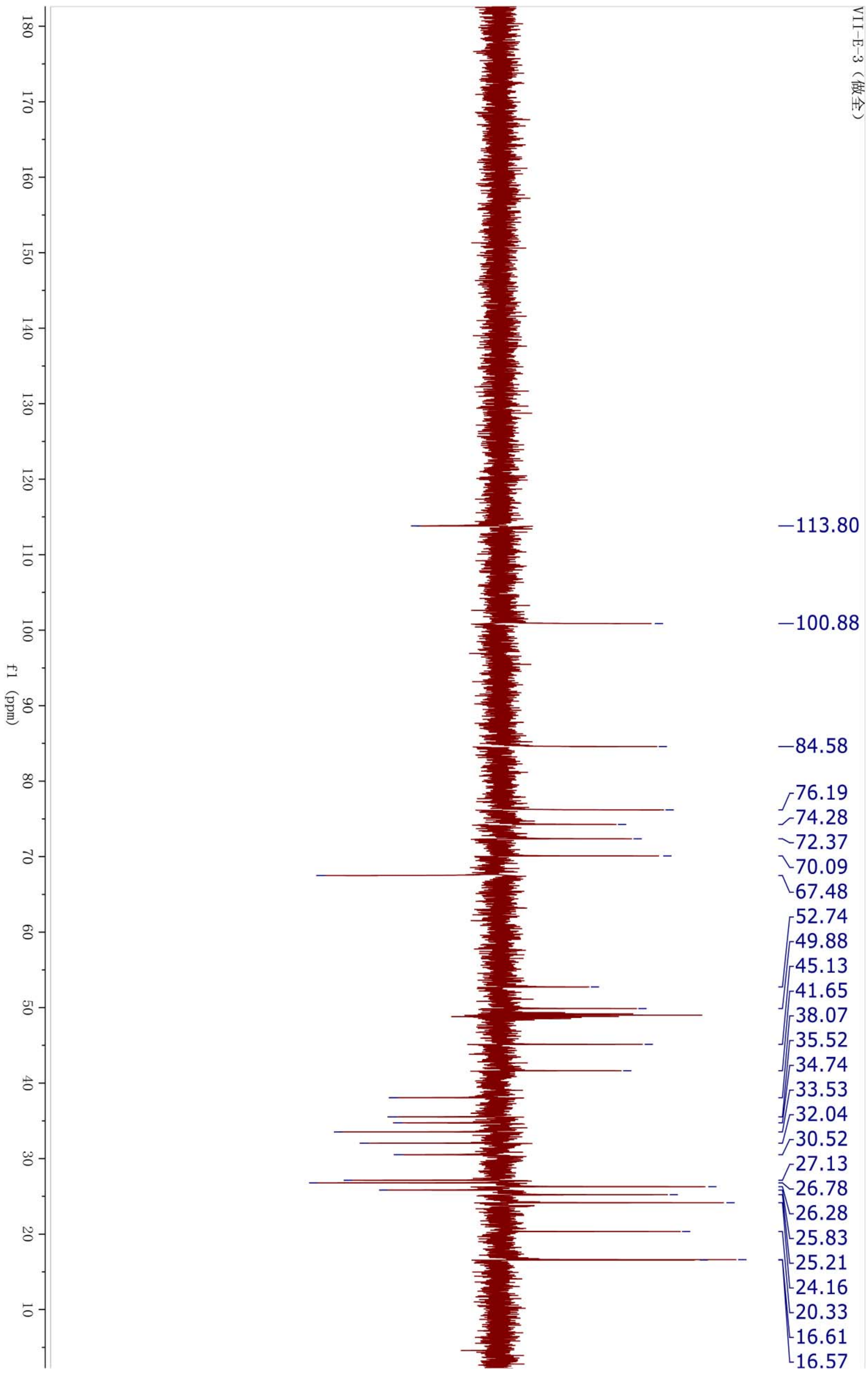

Figure S9. DEPT 135 spectrum (125 MHz, Methanol-d_4) of compound **1**.

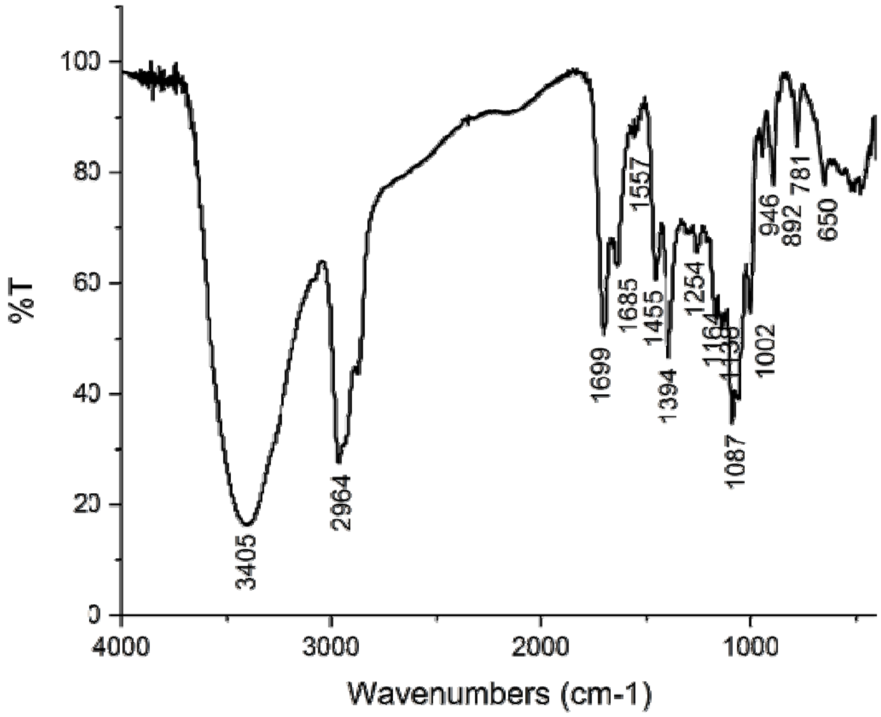

Figure S10. IR (KBr disc) spectrum of compound **1**.

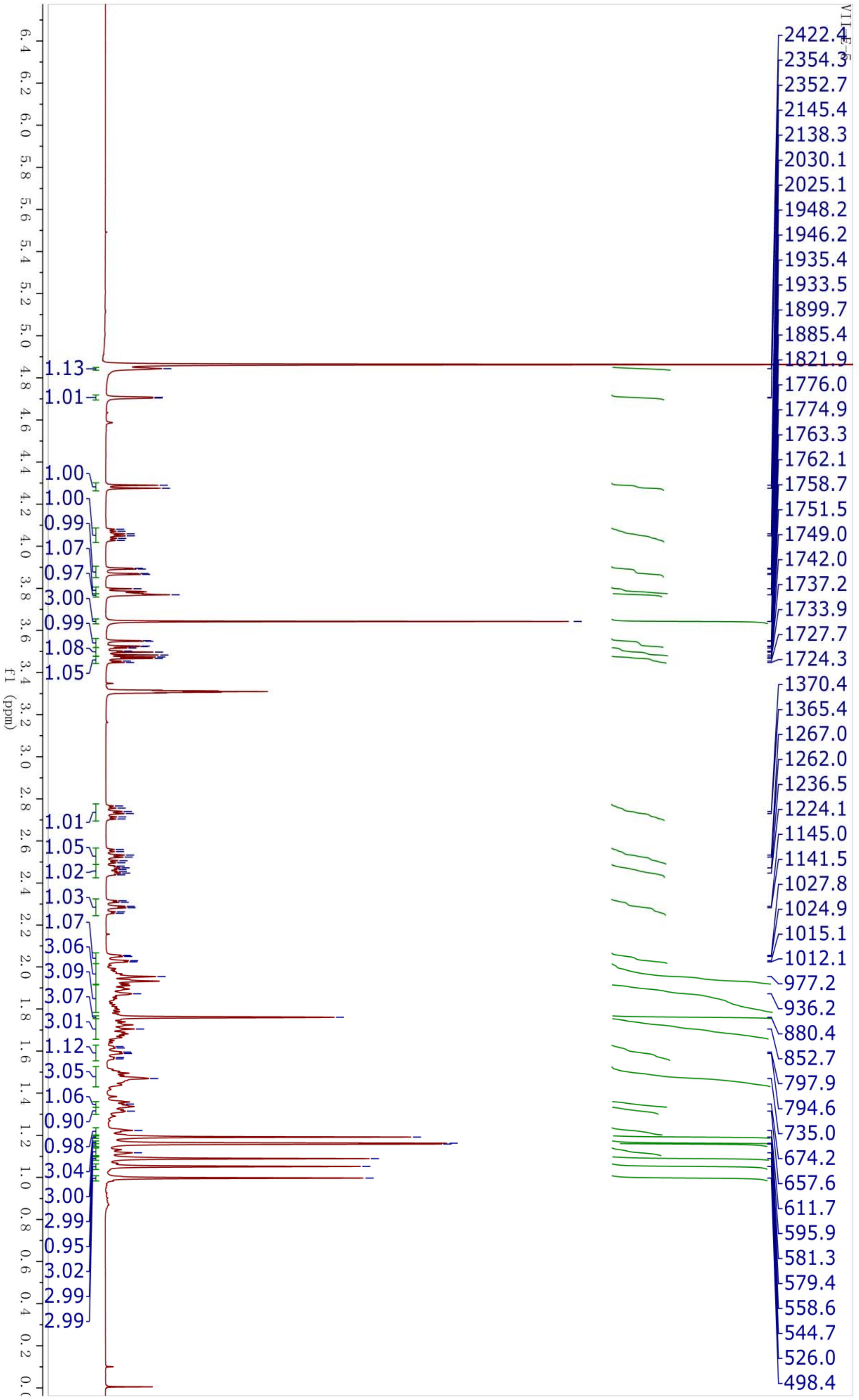

Figure S11. ^{1}H NMR spectrum (500 MHz, Methanol-d_4) of compound **2**.

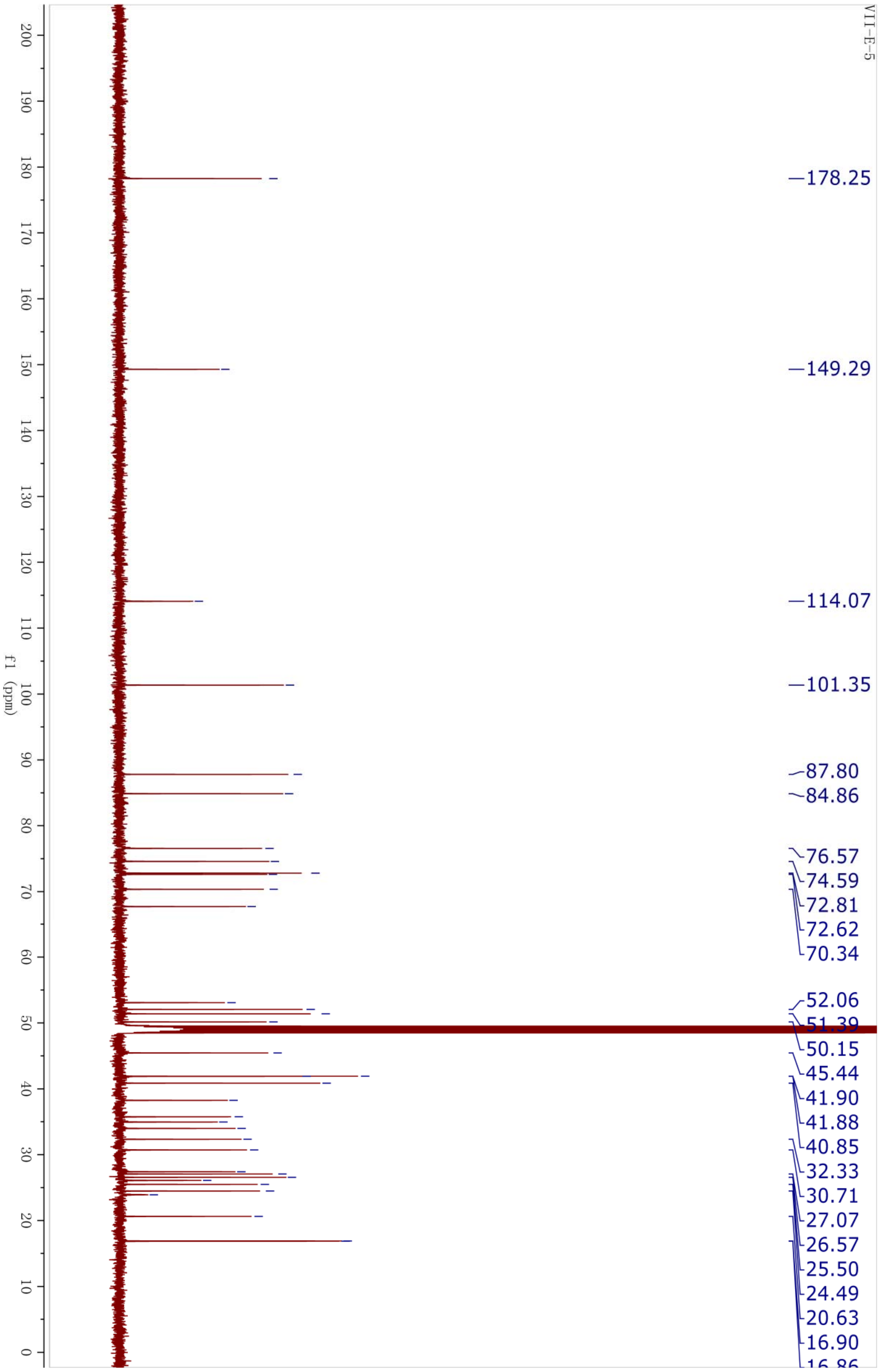

Figure S12. ^{13}C NMR spectrum (125 MHz, Methanol-d_4) of compound **2**.

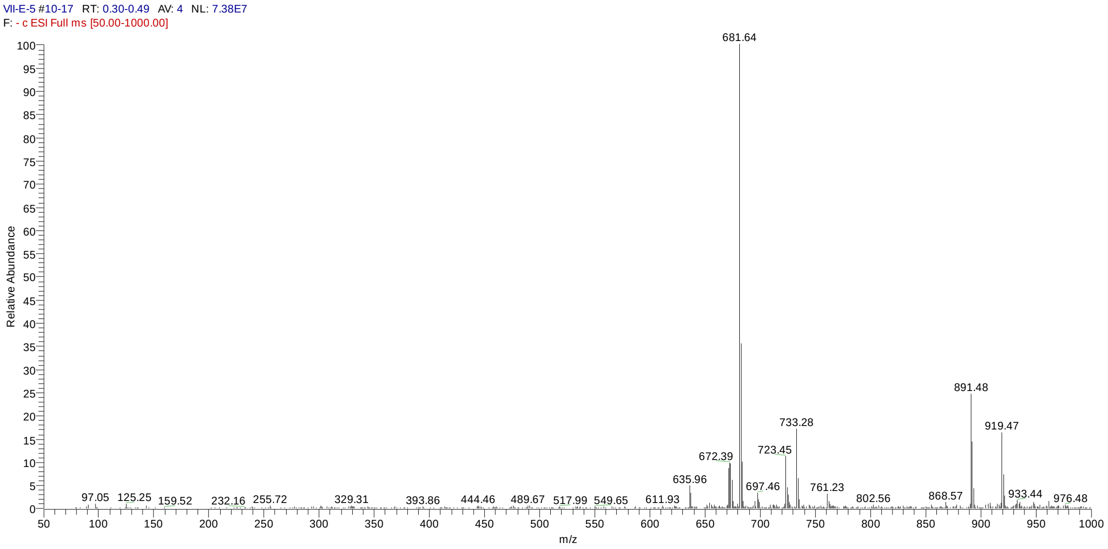

Figure S13. ESI MS (negative mode) spectrum of compound **2**.

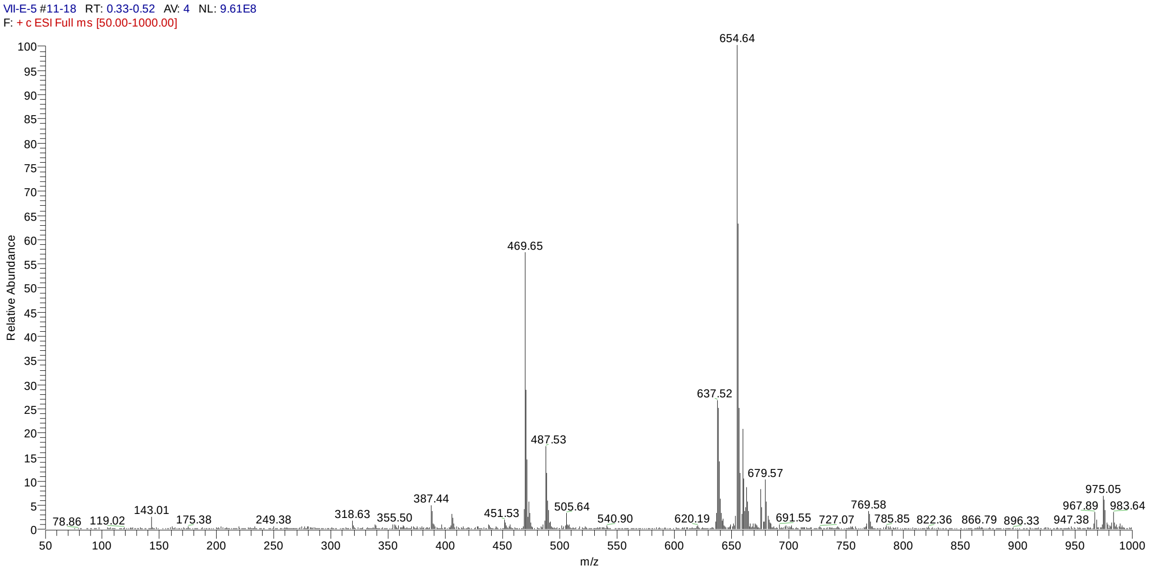

Figure S14. ESI MS (positive mode) spectrum of compound **2**.

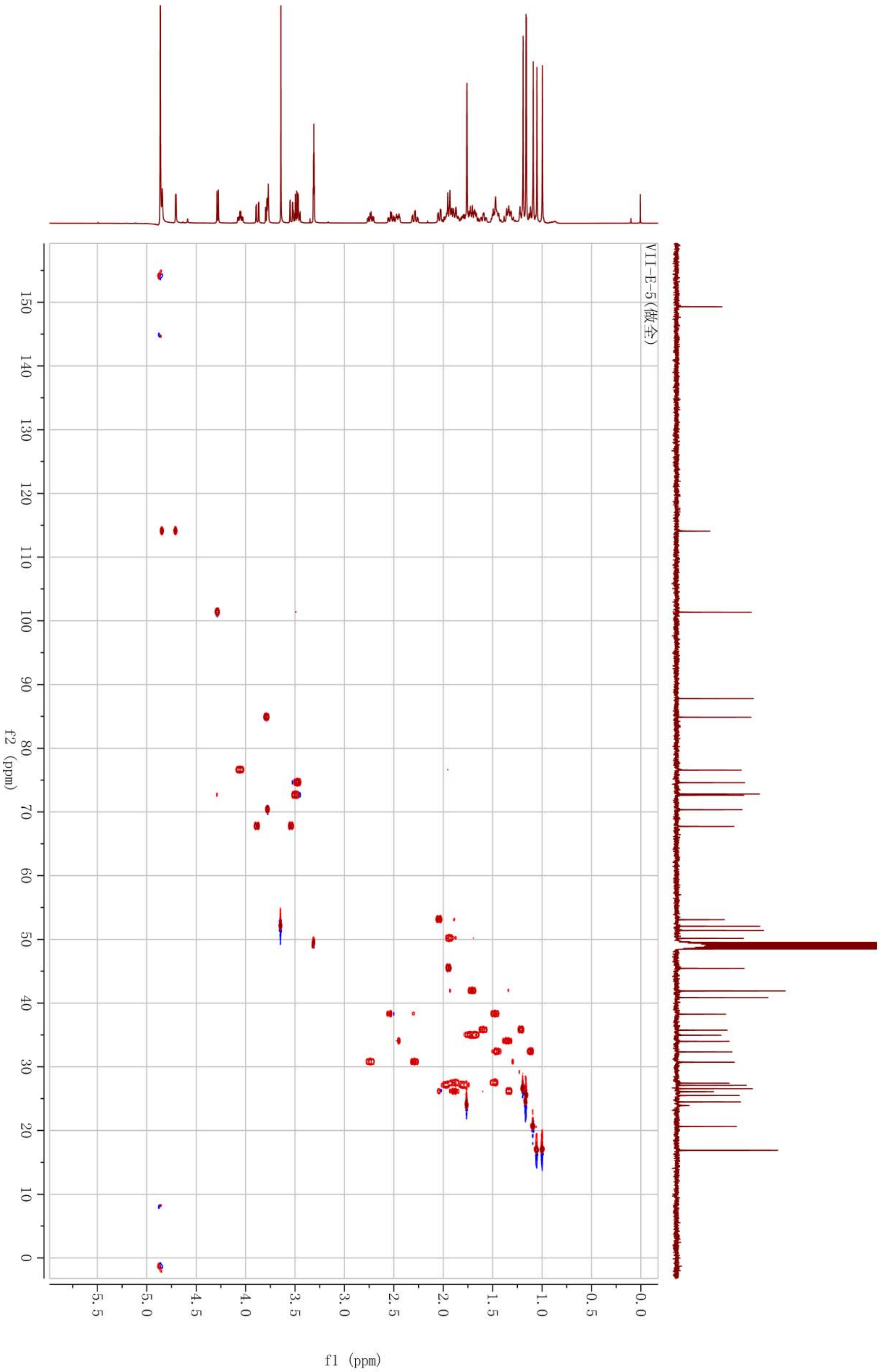

Figure S15. HMQC spectrum (500 MHz, Methanol-d_4) of compound **2**.

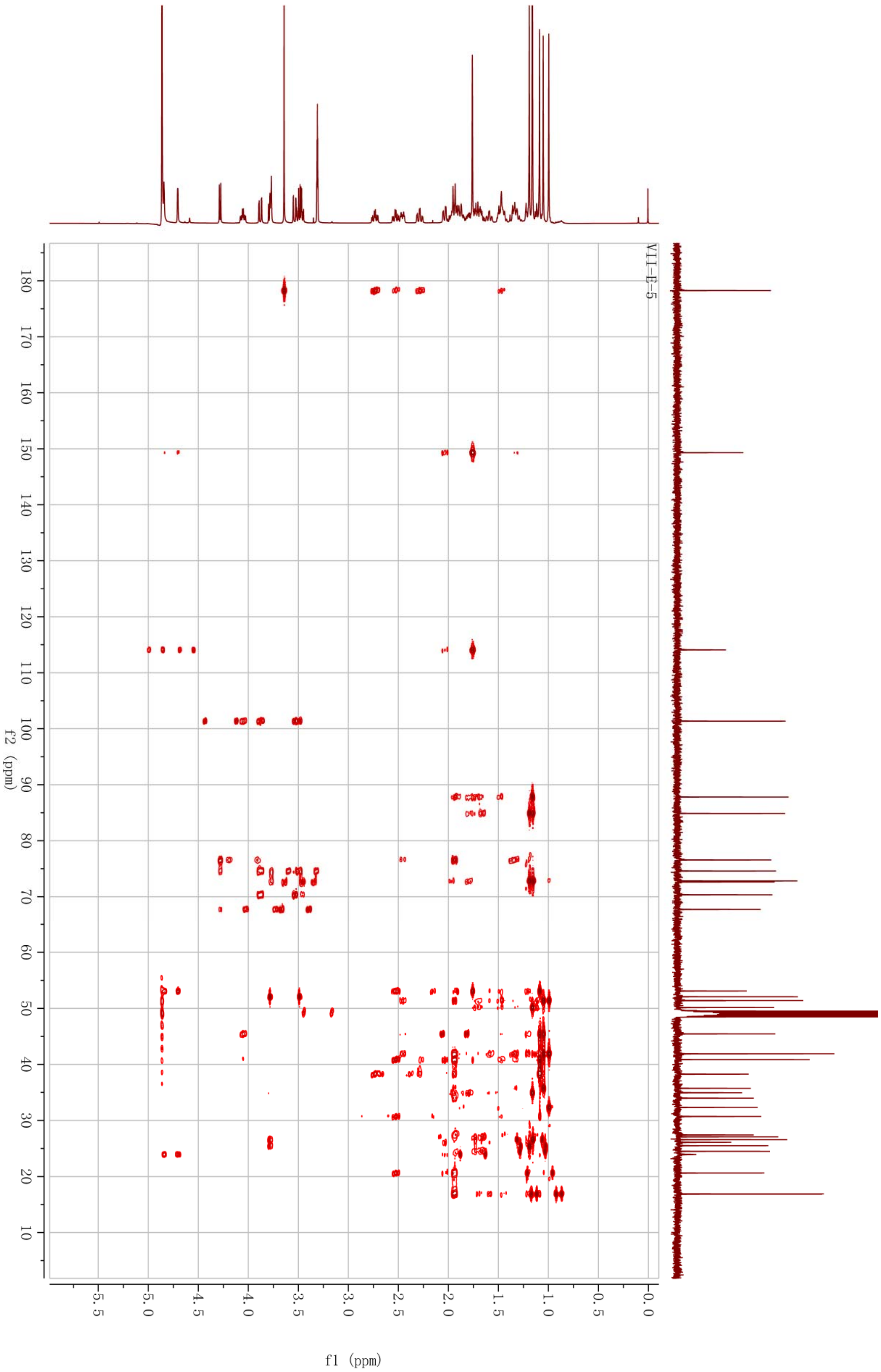

Figure S16. HMBC spectrum (500 MHz, Methanol-d_4) of compound **2**.

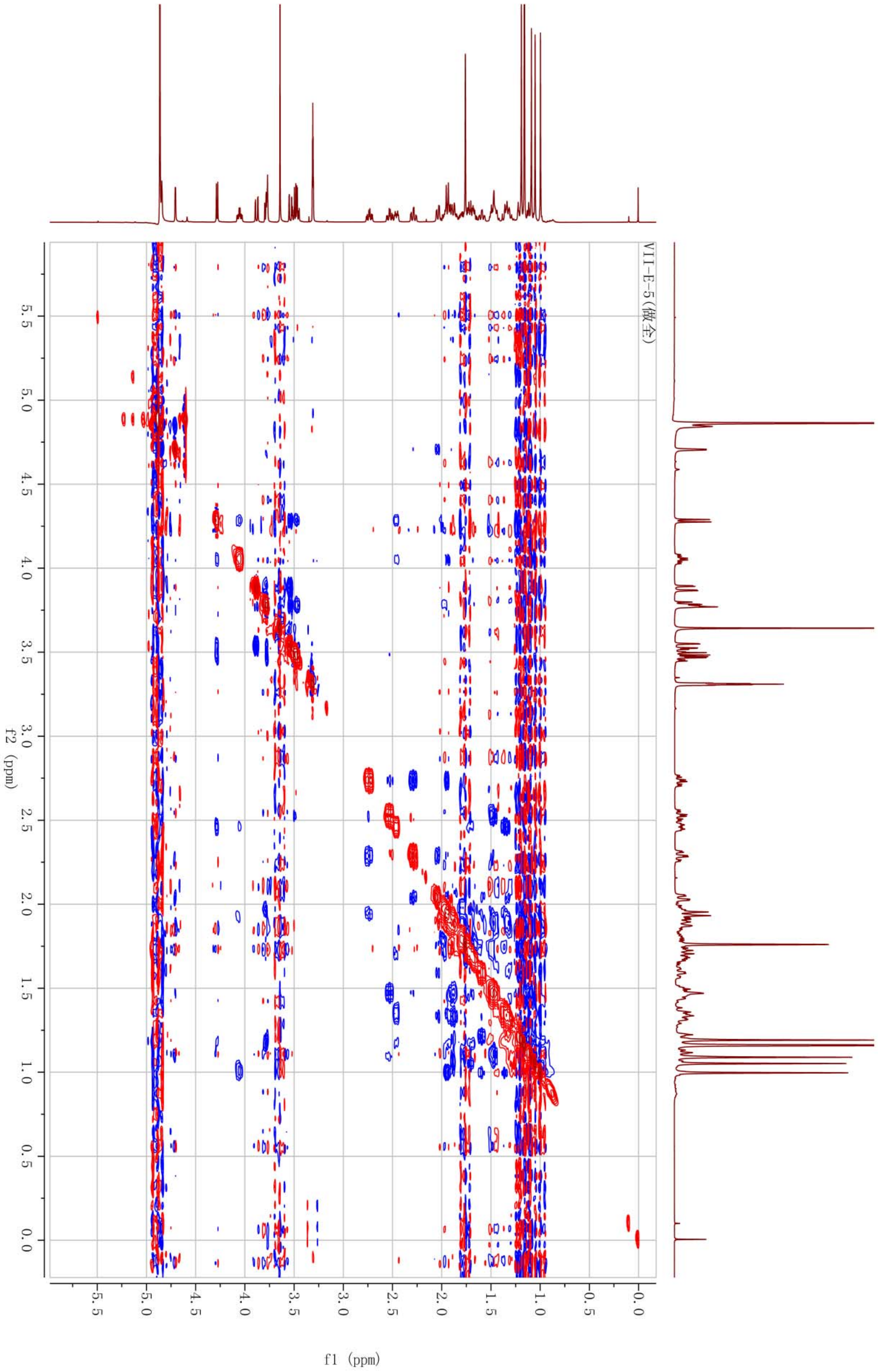

Figure S17. NOESY spectrum (500 MHz, Methanol-d_4) of compound **2**.

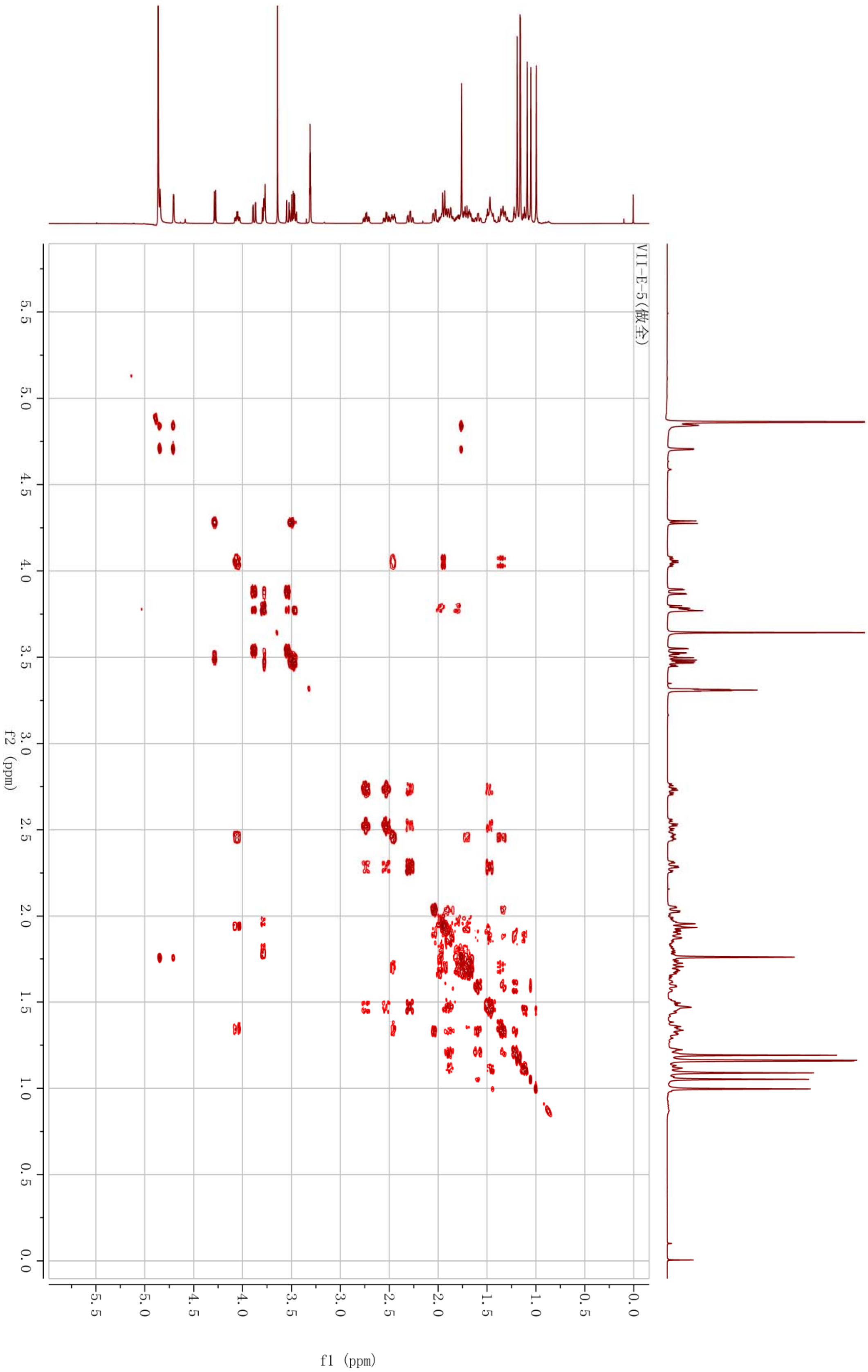

Figure S18. ^{1}H-^{1}H COSY spectrum (500 MHz, Methanol-d_4) of compound **2**.

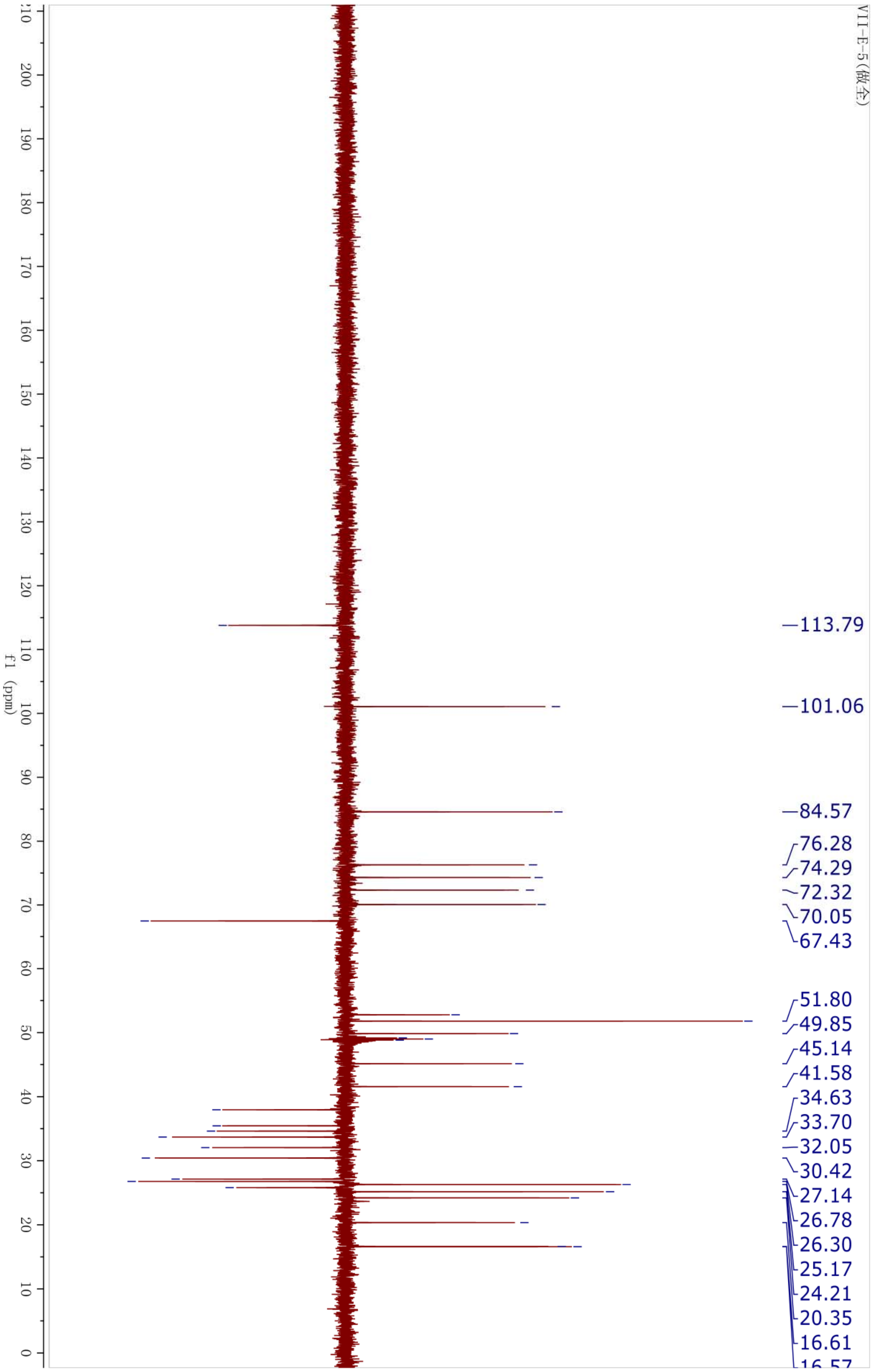

Figure S19. DEPT 135 spectrum (125 MHz, Methanol-d_4) of compound **2**.

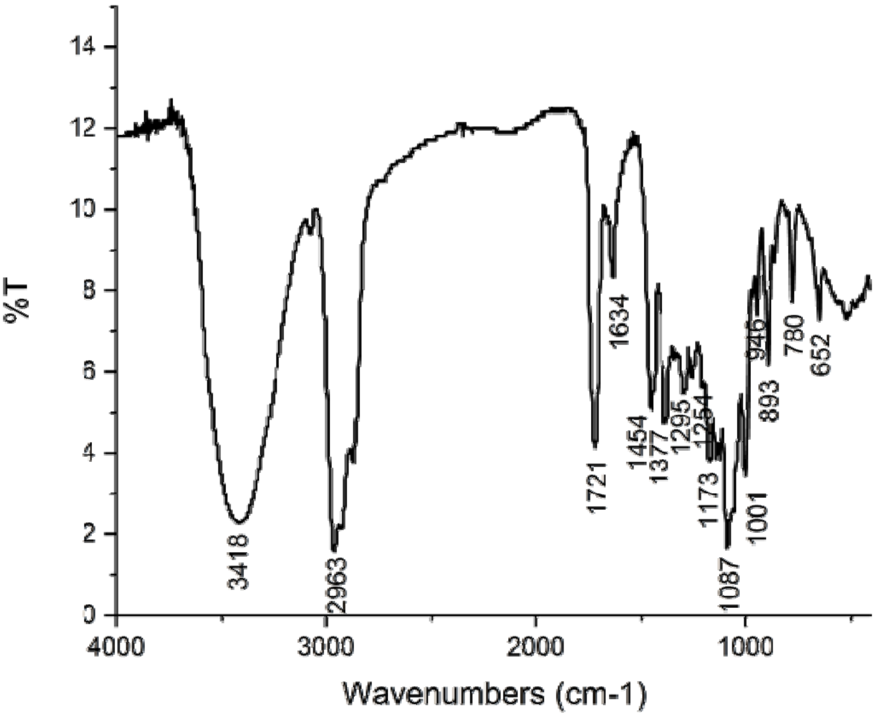

Figure S20. IR (KBr disc) spectrum of compound **2**.

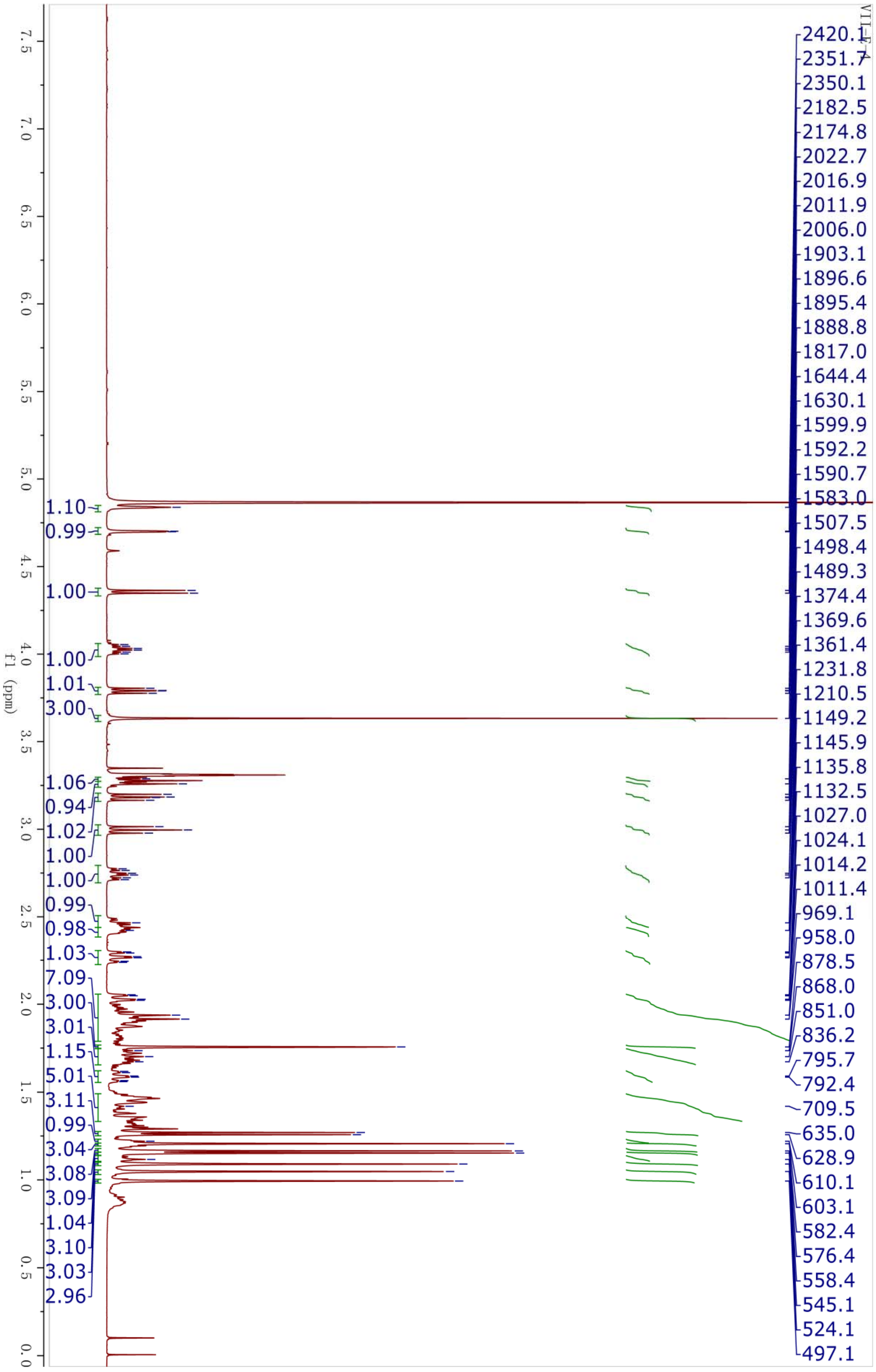

Figure S21. ^{1}H NMR spectrum (500 MHz, Methanol-d_4) of compound **3**.

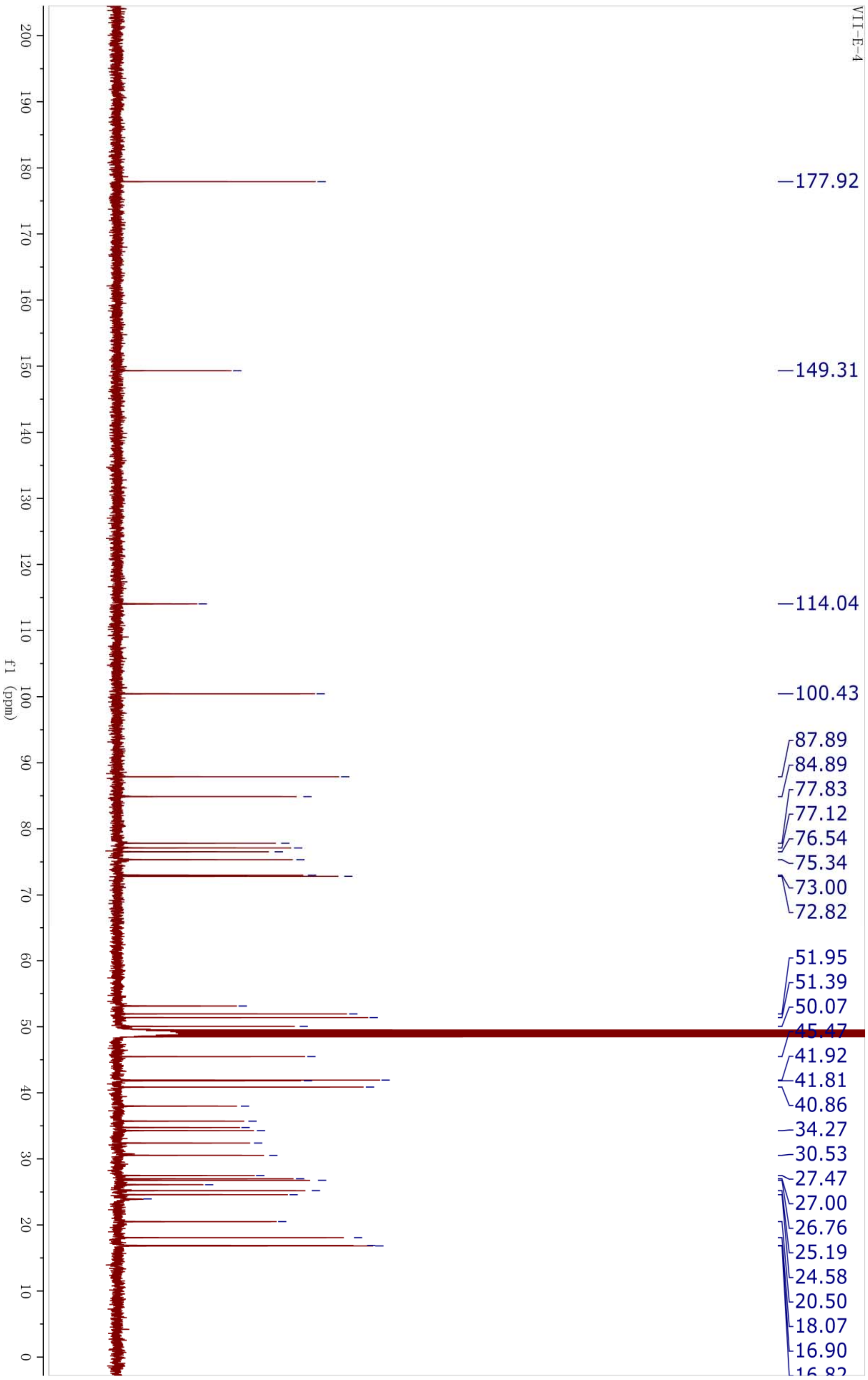

Figure S22. ^{13}C NMR spectrum (125 MHz, Methanol-d_4) of compound **3**.

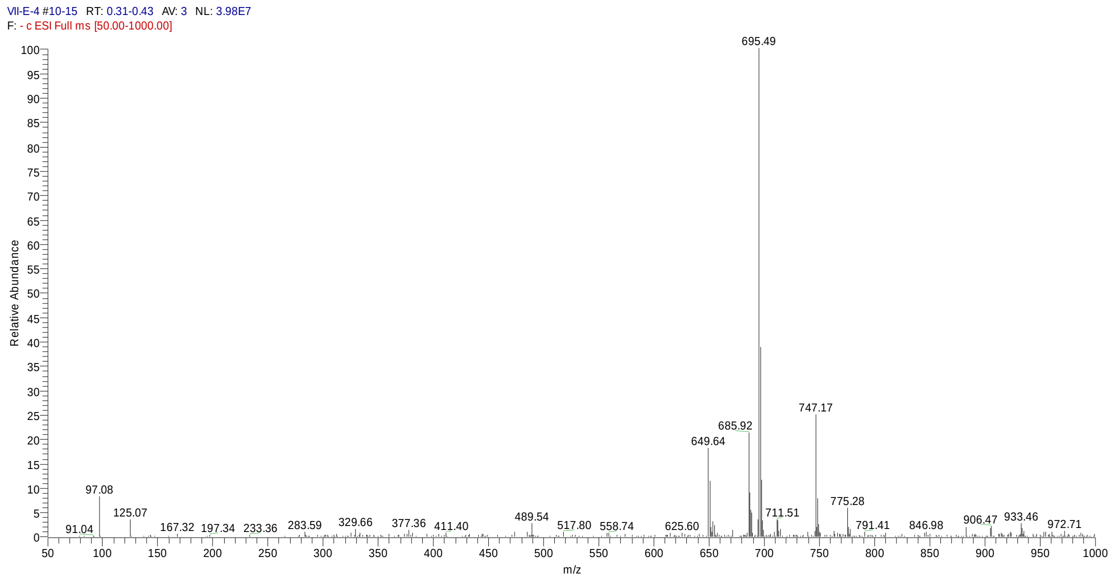

Figure S23. ESI MS (negative mode) spectrum of compound **3**.

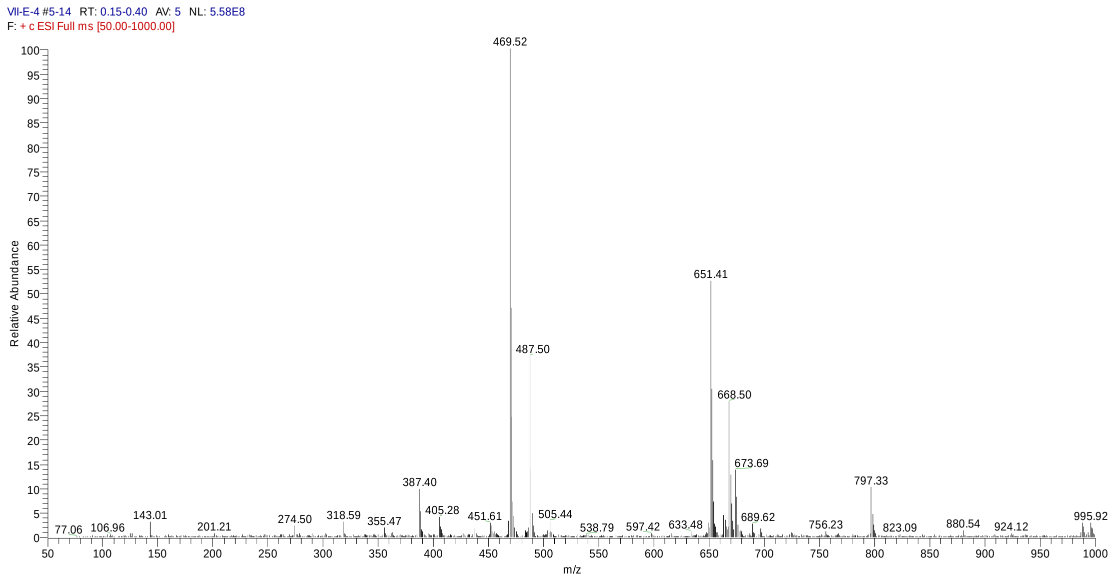

Figure S24. ESI MS (positive mode) spectrum of compound **3**.

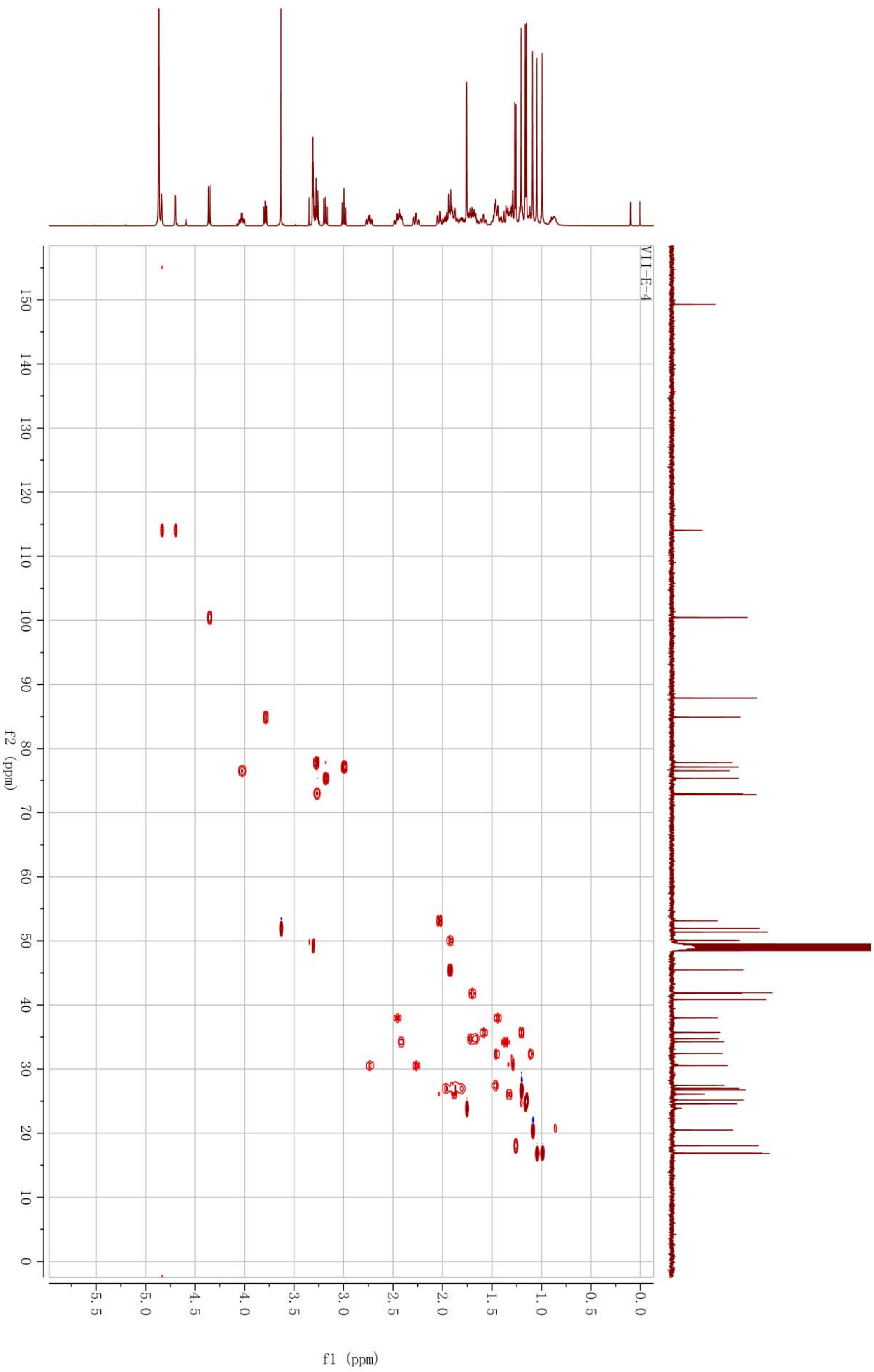

Figure S25. HMQC spectrum (500 MHz, Methanol-d_4) of compound **3**.

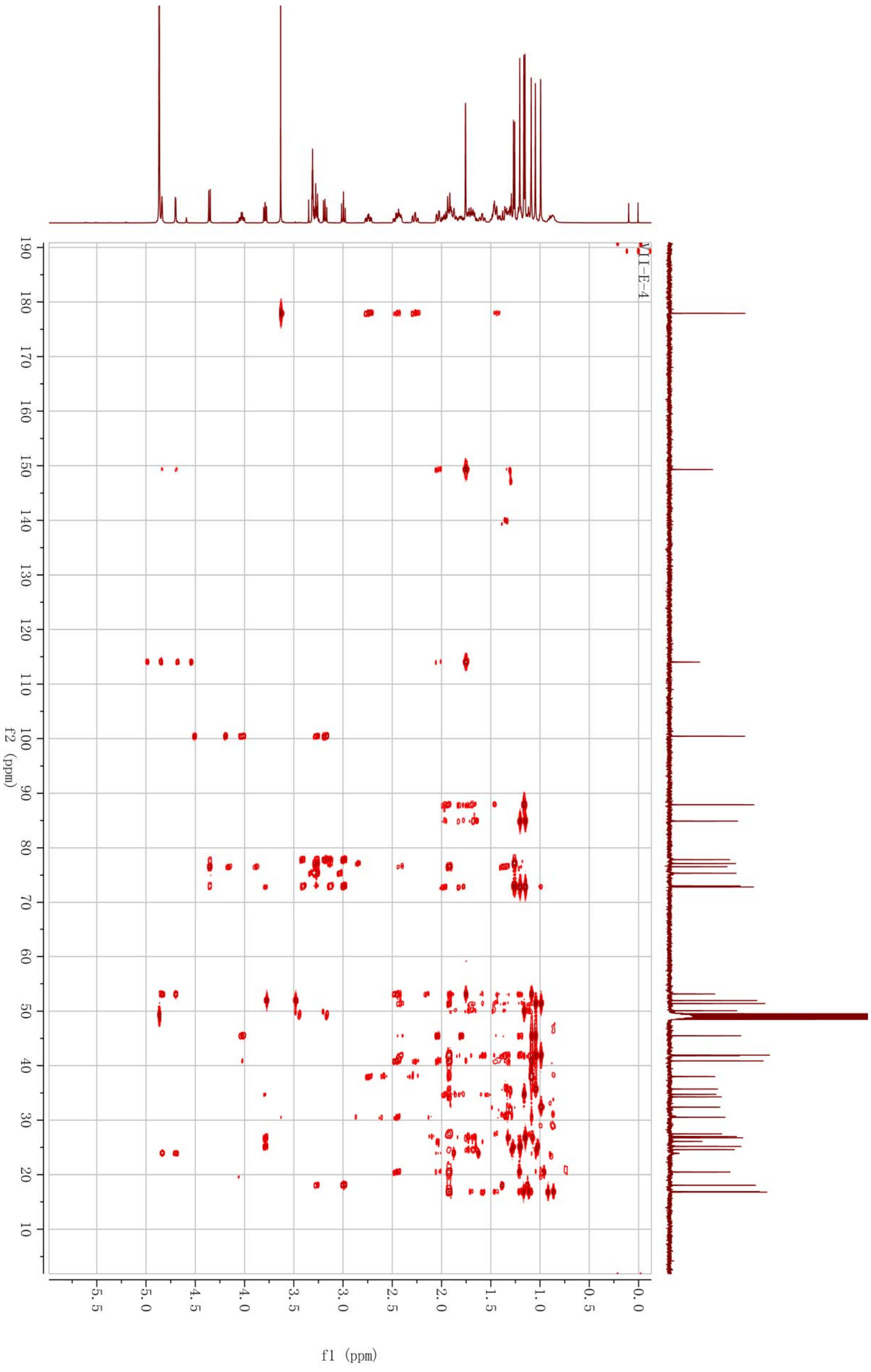

Figure S26. HMBC spectrum (500 MHz, Methanol-d_4) of compound **3**.

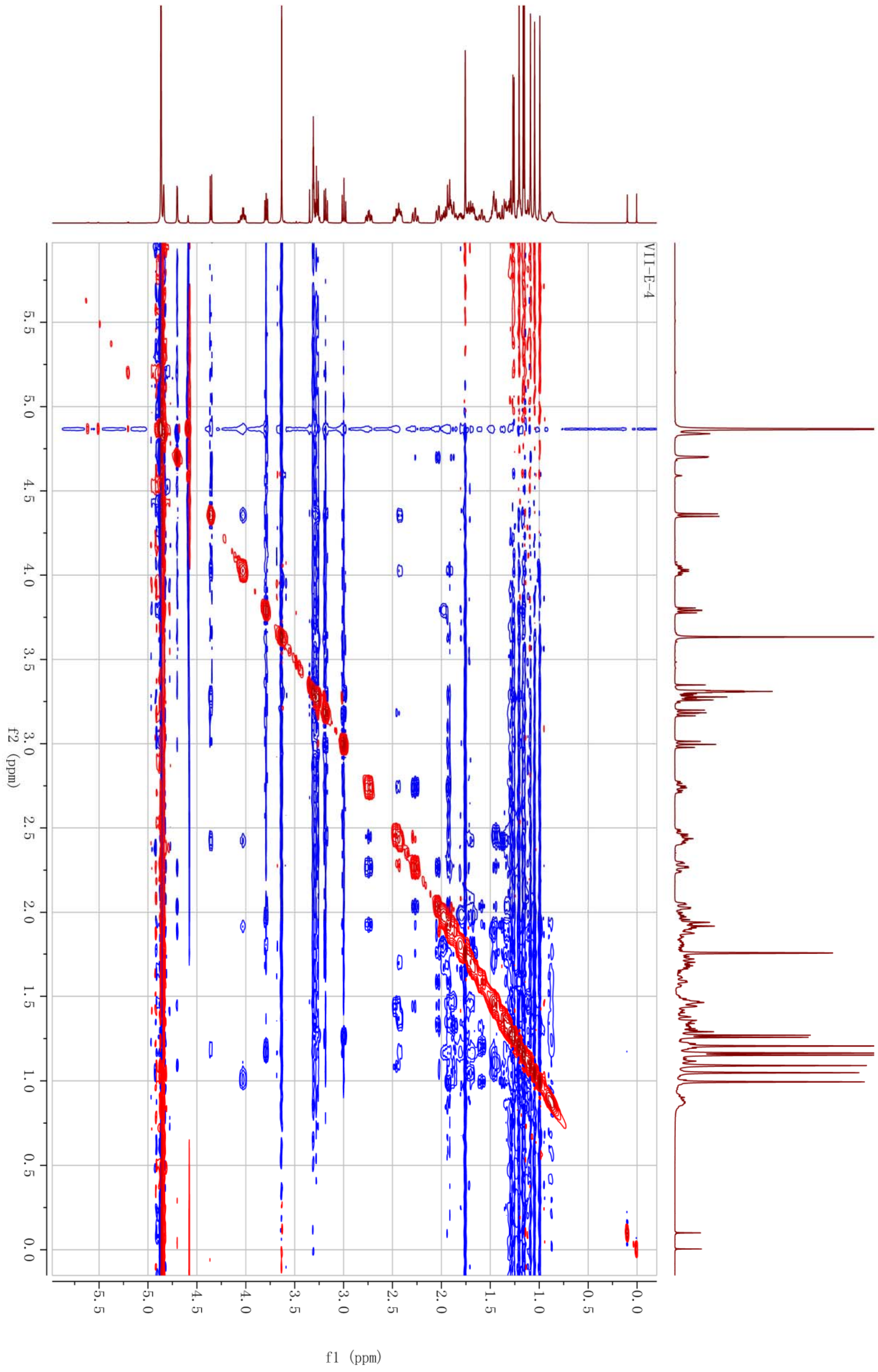

Figure S27. NOESY spectrum (500 MHz, Methanol-d_4) of compound **3**.

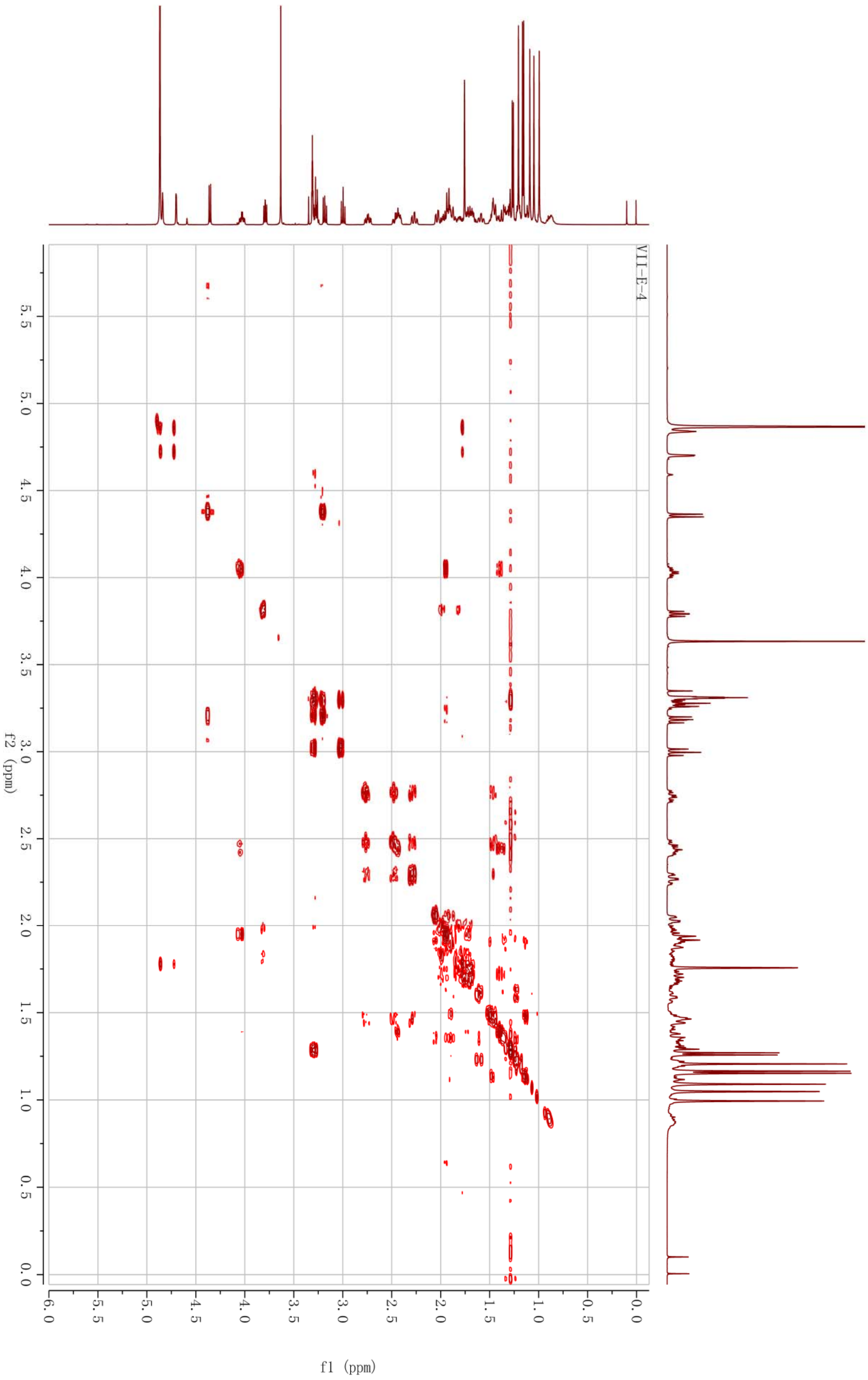

Figure S28. ^1H-^1H COSY spectrum (500 MHz, Methanol-d_4) of compound **3**.

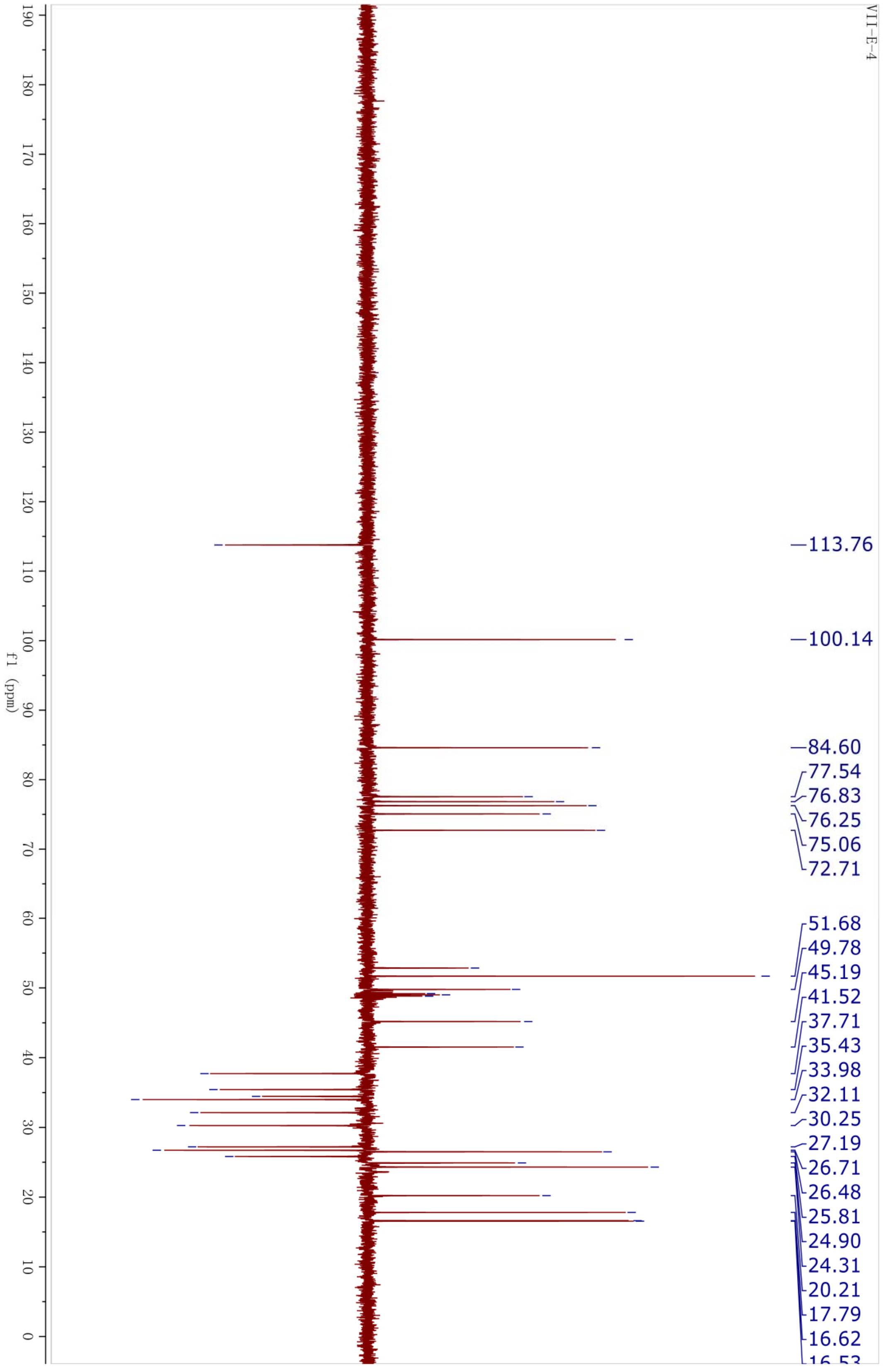

Figure S29. DEPT 135 spectrum (125 MHz, Methanol-d_4) of compound **3**.

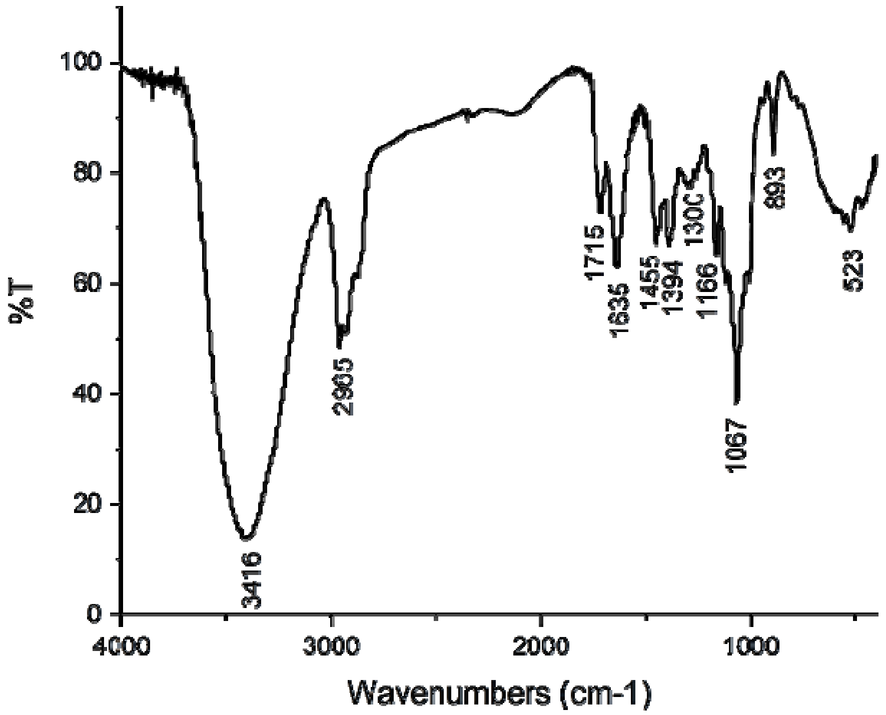

Figure S30. IR (KBr disc) spectrum of compound **3**.

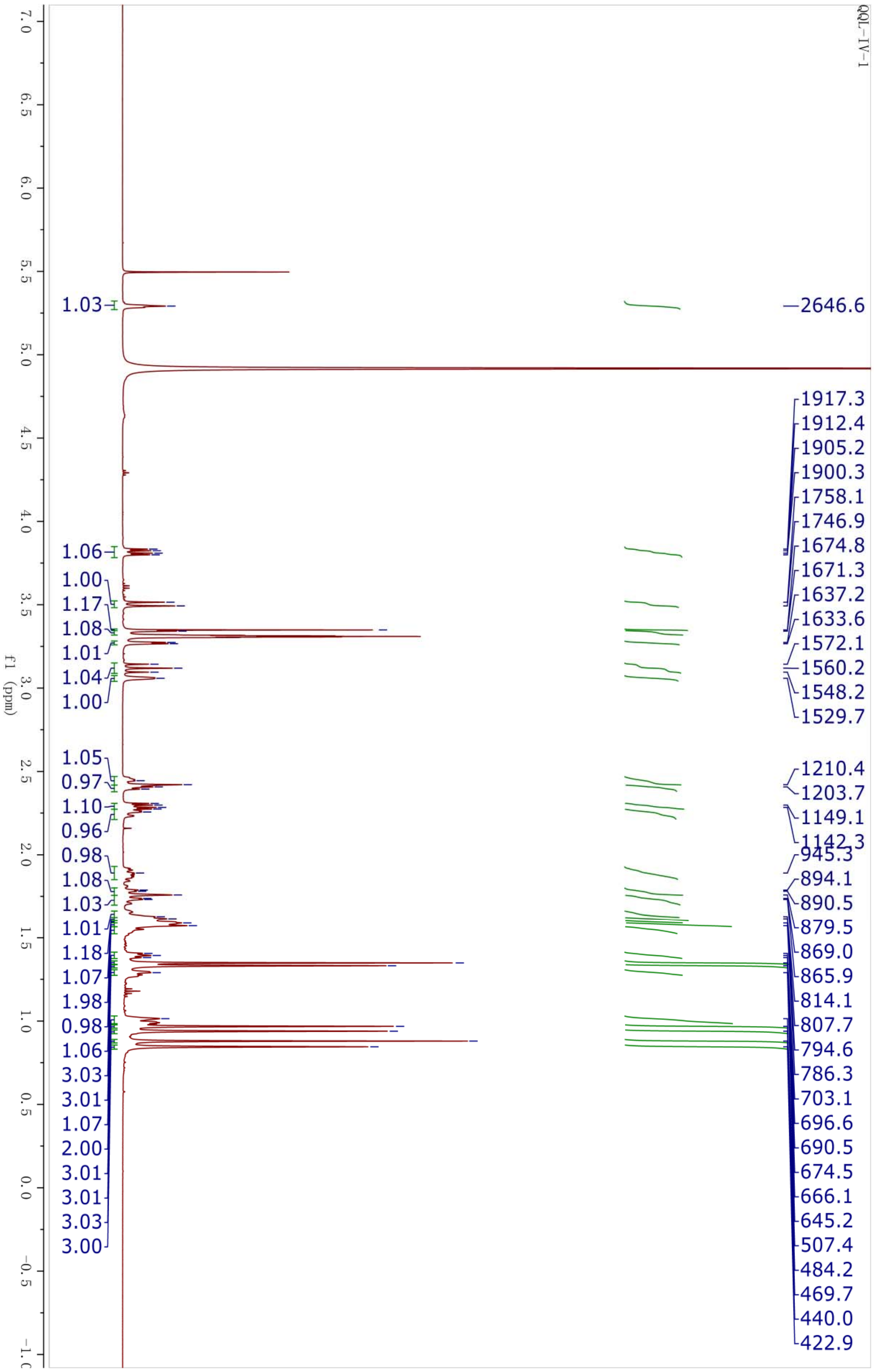

Figure S31. ^{1}H NMR spectrum (500 MHz, Methanol-d_4) of compound **4**.

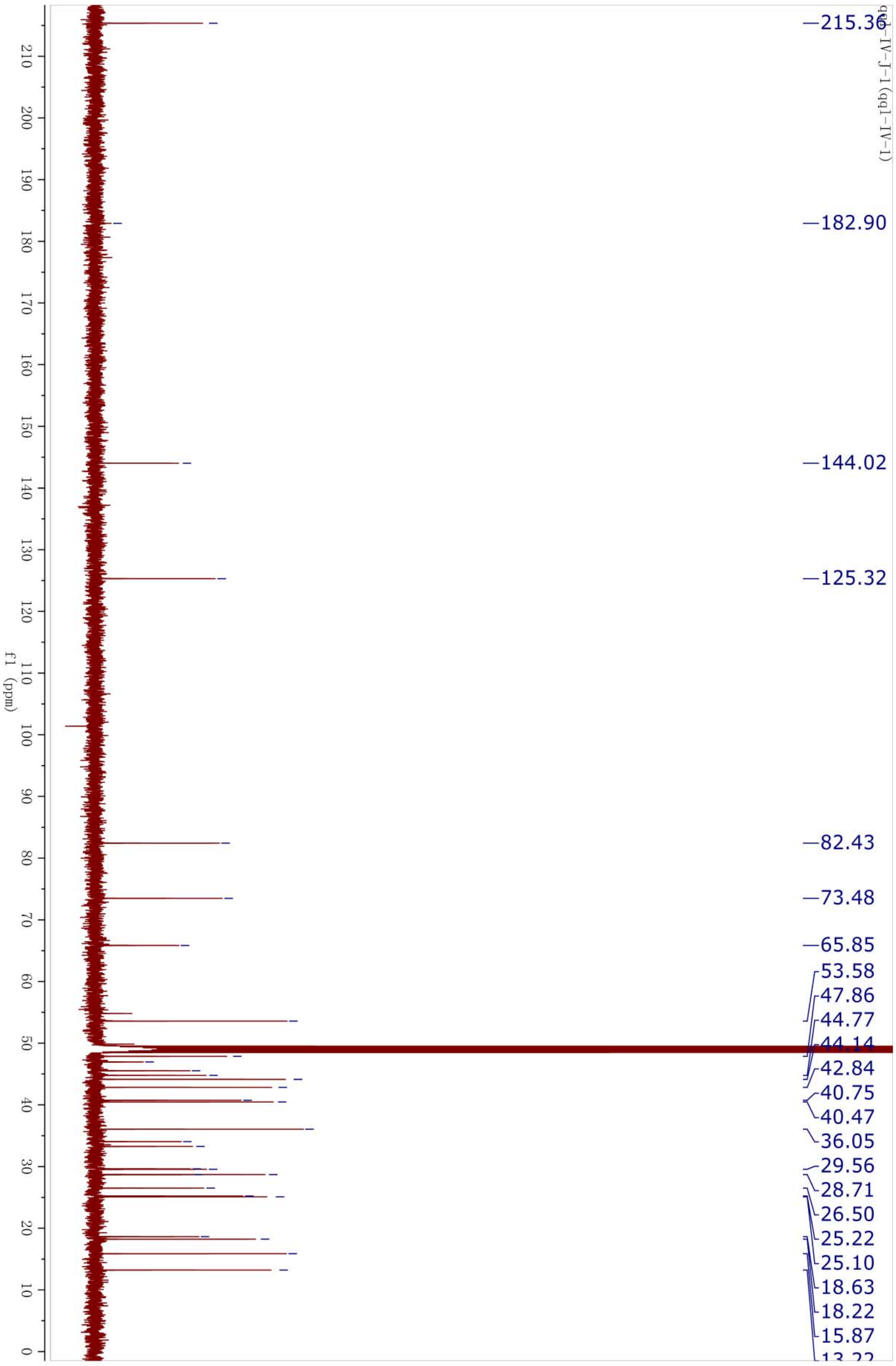

Figure S32. ^{13}C NMR spectrum (125 MHz, Methanol-d_4) of compound **4**.

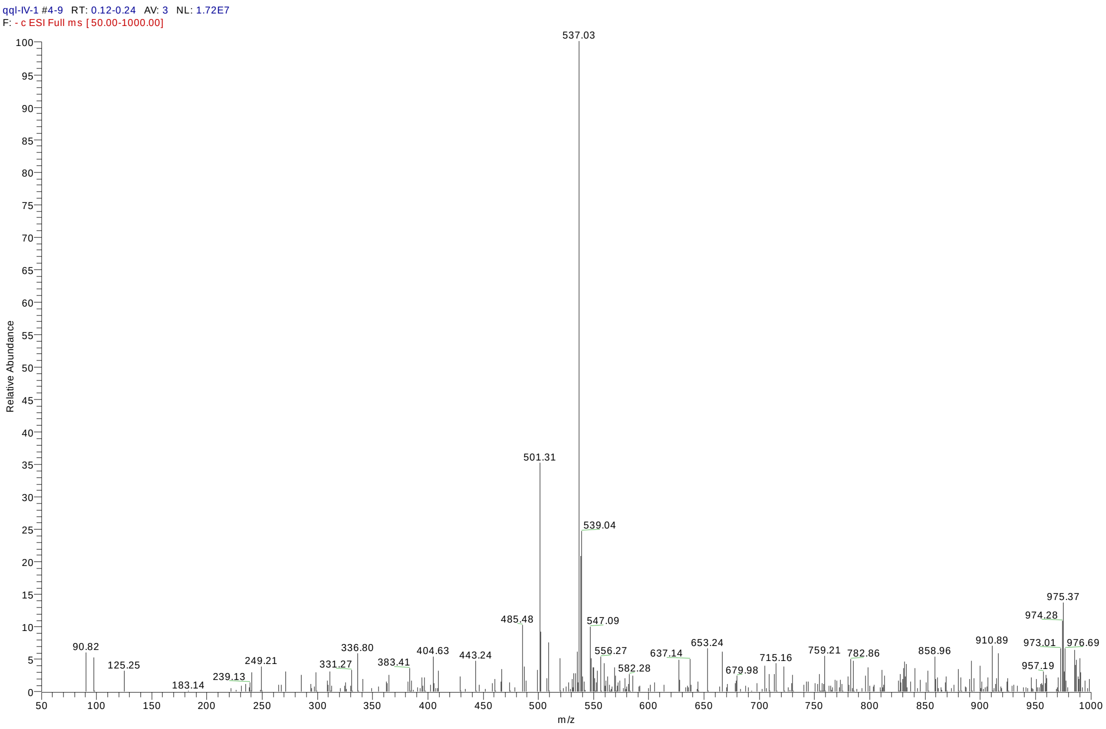

Figure S33. ESI MS (negative mode) spectrum of compound **4**.

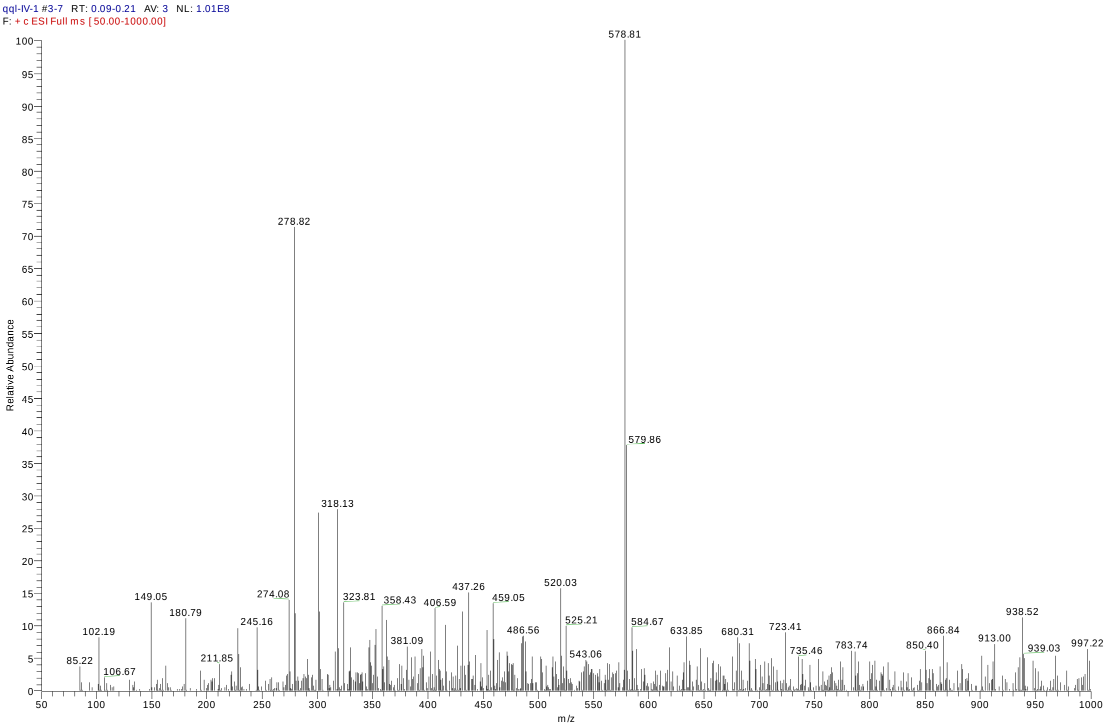

Figure S34. ESI MS (positive mode) spectrum of compound **4**.

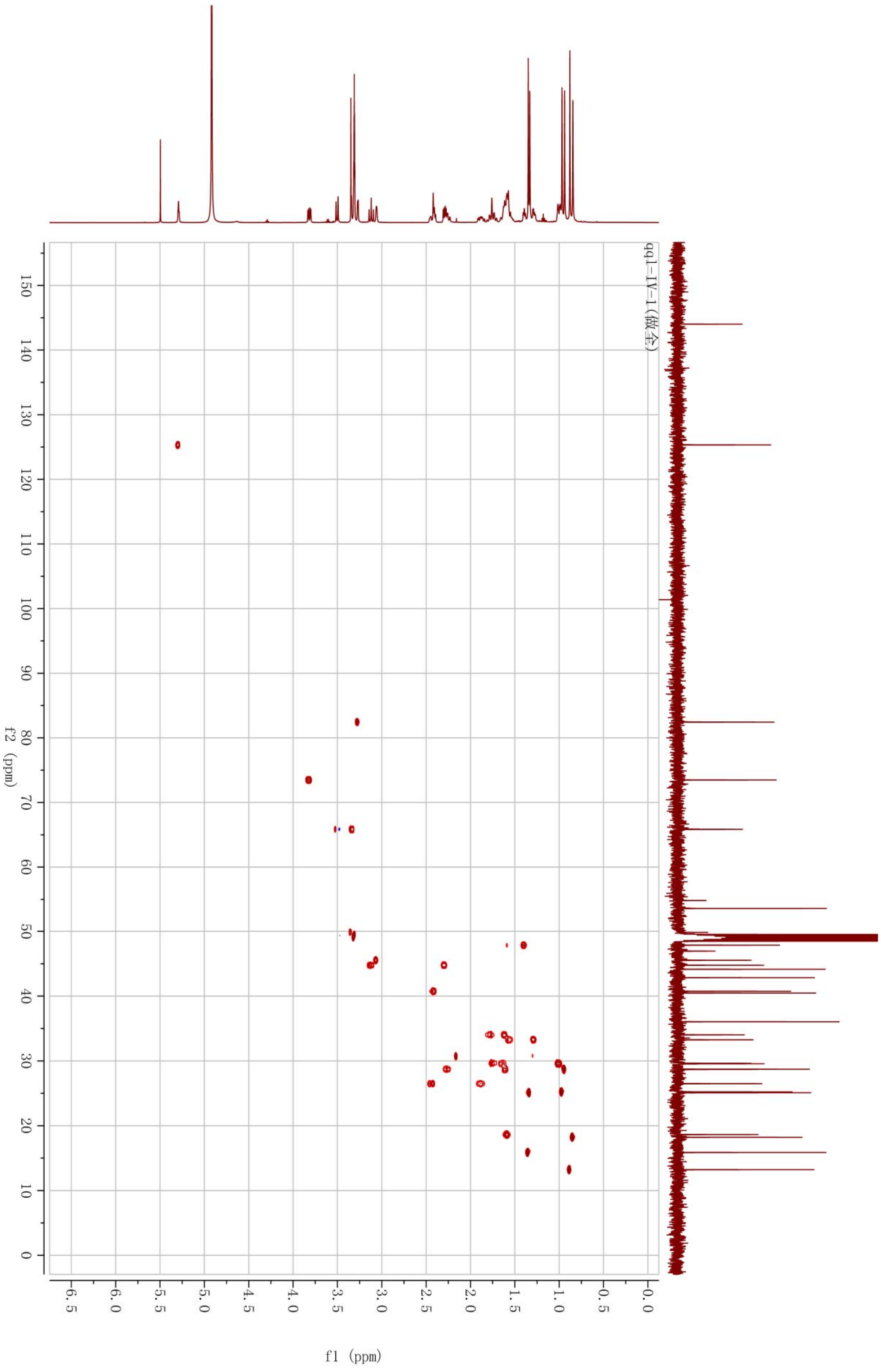

Figure S35. HMQC spectrum (500 MHz, Methanol-d_4) of compound **4**.

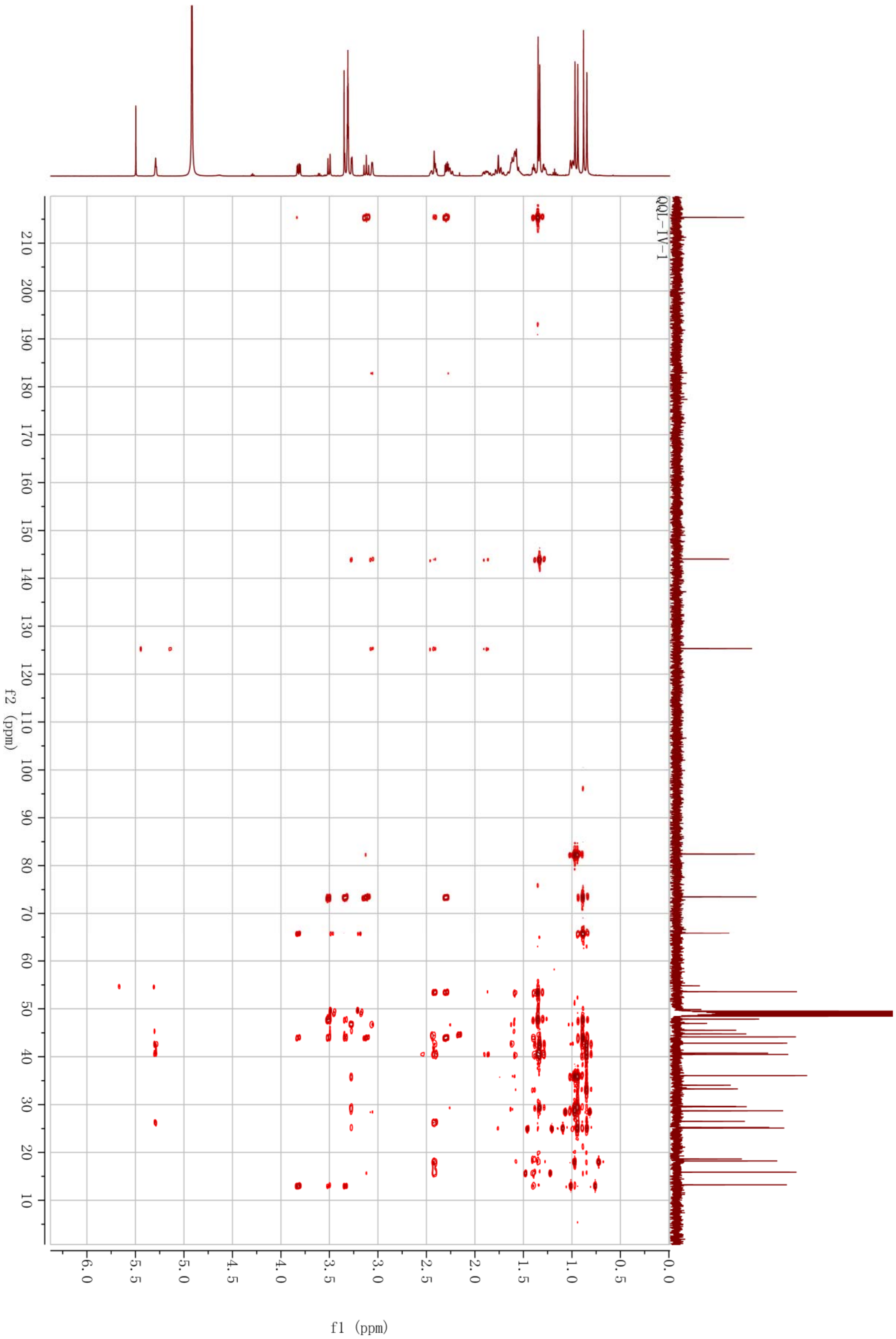

Figure S36. HMBC spectrum (500 MHz, Methanol-d_4) of compound **4**.

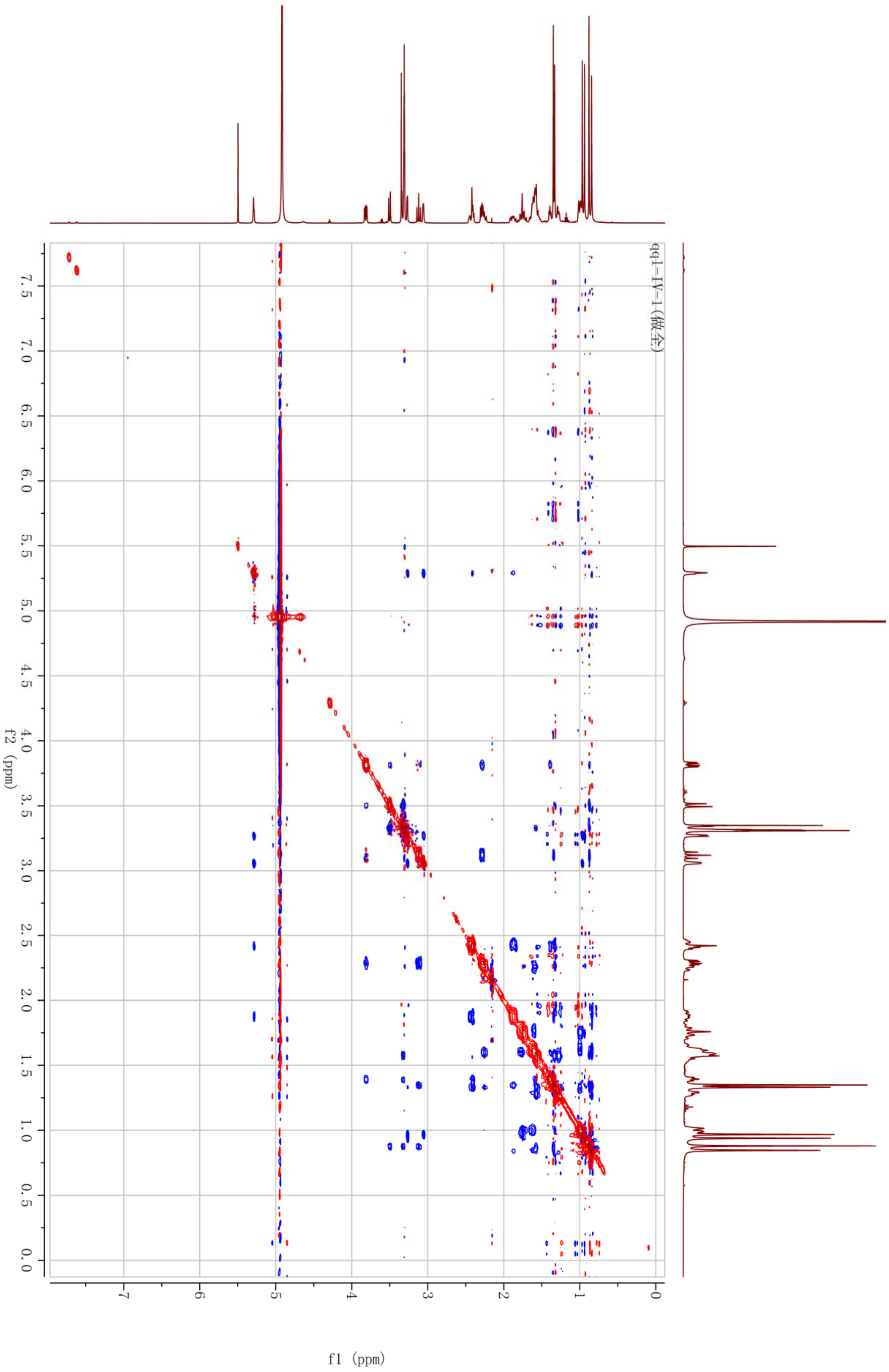

Figure S37. NOESY spectrum (500 MHz, Methanol-d_4) of compound **4**.

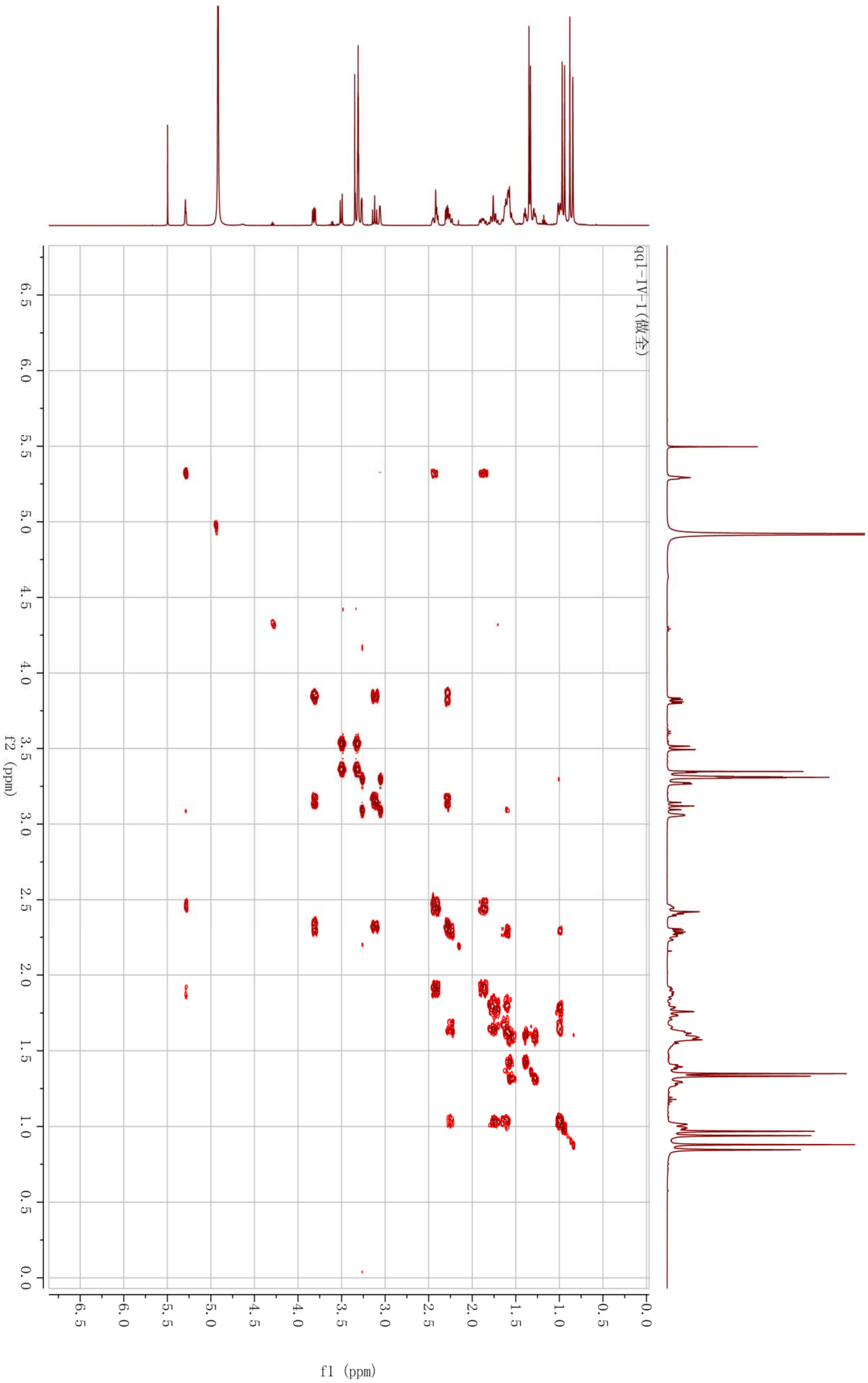

Figure S38. ^1H-^1H COSY spectrum (500 MHz, Methanol-d_4) of compound **4**.

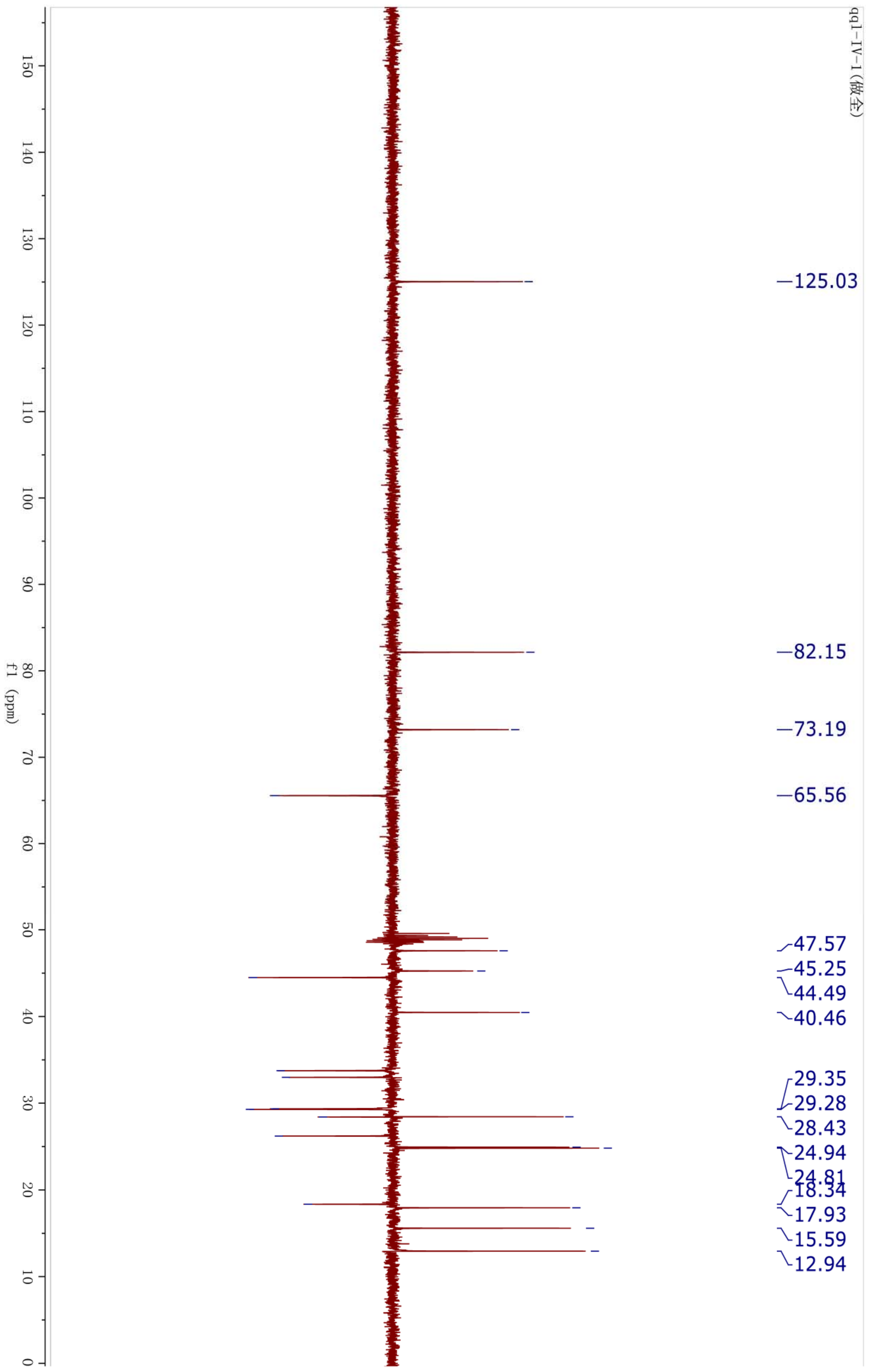

Figure S39. DEPT 135 spectrum (125 MHz, Methanol-d_4) of compound **4**.

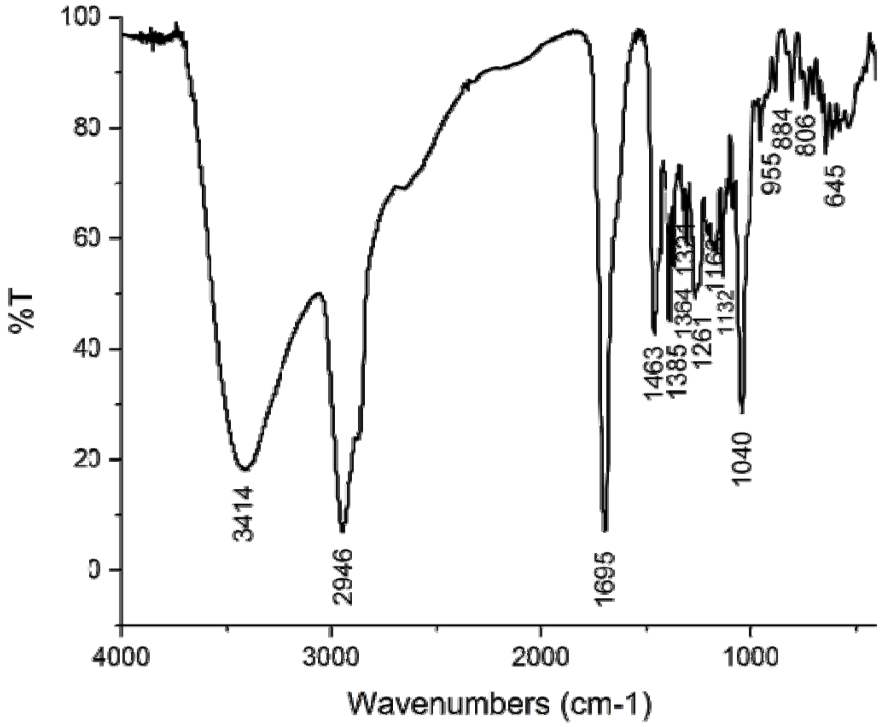

Figure S40. IR (KBr disc) spectrum of compound **4**.

Table S1. Structures of known compounds.

comp.	name	structure	molecular formula
**5**	arjunolic acid	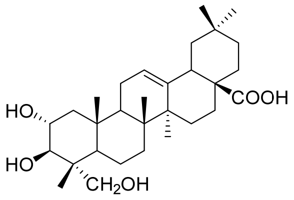	$C_{30}H_{48}O_5$
**6**	cyclocaric acid B	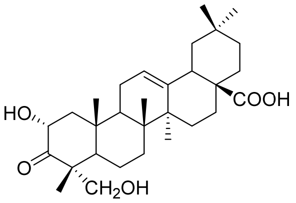	$C_{30}H_{46}O_5$
**7**	1*α*, 3*β*-dihydroxy-olean-12-en-28-oic acid	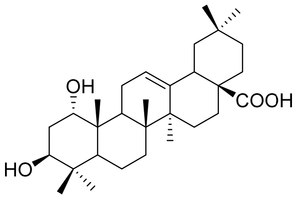	$C_{30}H_{48}O_4$
**8**	punicaone	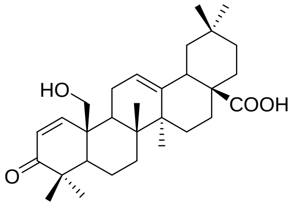	$C_{30}H_{44}O_4$
**9**	olean-12-en-1*β*,3*β*,28-triol	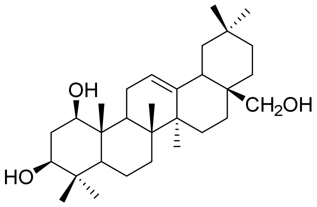	$C_{30}H_{50}O_3$
**10**	ursolic acid	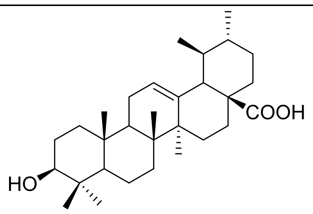	$C_{30}H_{48}O_3$
**11**	asiatic acid	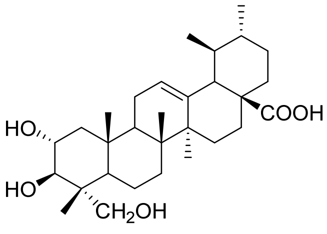	$C_{30}H_{48}O_5$

**12**	cyclocarioside K	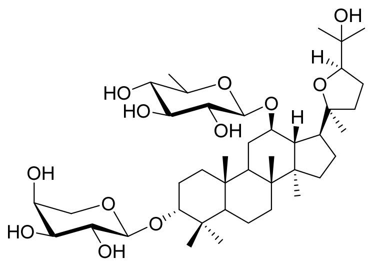	$C_{41}H_{70}O_{12}$
**13**	cyclocarioside H	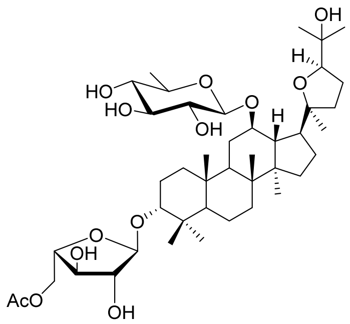	$C_{43}H_{72}O_{13}$
**14**	cyclocarioside I	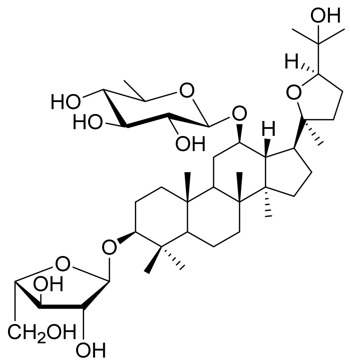	$C_{41}H_{70}O_{12}$
**15**	cyclocarioside B	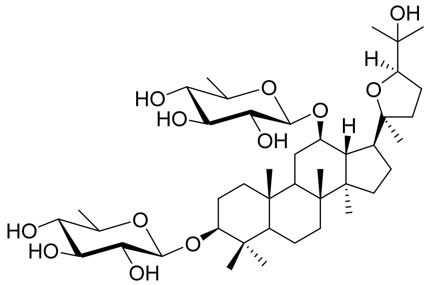	$C_{42}H_{72}O_{12}$

Table S2. Cytotoxicity of the isolates in C2C12 myotubes and 3T3-L1 adipocytes. *n* = 9.

	Cell viability (%)	
Compound (μM)	C2C12 myotubes	3T3-L1 adipocytes
DMSO	100.05 ± 1.77	100.14 ± 1.22
RSV (5)	95.96 ± 2.15	100.65 ± 1.90
**1** (10)	93.61 ± 2.42	99.92 ± 2.63
**2** (2)	104.52 ± 2.78	98.23 ± 3.36
**3** (2)	100.32 ± 1.70	100.38 ± 1.91
**4** (10)	106.49 ± 2.58	99.68 ± 2.86
**5** (10)	92.29 ± 5.23	97.75 ± 1.43
**6** (10)	93.92 ± 1.28	98.88 ± 2.69
**7** (10)	98.64 ± 1.69	100.03 ± 1.19
**8** (10)	97.39 ± 1.07	100.54 ± 3.64
**9** (10)	95.76 ± 2.57	98.19 ± 1.27
**10** (10)	96.24 ± 7.70	99.61 ± 3.43
**11** (10)	95.28 ± 4.98	98.18 ± 1.23
**12** (10)	100.26 ± 1.92	99.25 ± 3.47
**13** (2)	96.99 ± 2.75	100.86 ± 1.08
**14** (10)	96.05 ± 6.66	101.71 ± 0.96
**15** (10)	98.79 ± 2.55	98.68 ± 2.11
